# Pik3ip1 mediates thyroid hormone-dependent regulation of the PI3K/Akt/mTOR axis in muscle atrophy

**DOI:** 10.1016/j.molmet.2026.102413

**Published:** 2026-07-06

**Authors:** Annarita Nappi, Serena Sagliocchi, Federica Restolfer, Caterina Miro, Lucia Acampora, Giovanna Giuseppina Altobelli, Monica Dentice, Annunziata Gaetana Cicatiello

**Affiliations:** 1 Department of Clinical Medicine and Surgery, University of Naples Federico II, 80131, Naples, Italy; 2 CEINGE – Biotecnologie Avanzate Scarl, Naples, Italy

**Keywords:** Thyroid hormone, Skeletal muscle, Muscle atrophy, PI3K/Akt/mTOR pathway, Metabolic regulation, Denervation

## Abstract

Skeletal muscle atrophy is driven by an imbalance between anabolic and catabolic signaling pathways, often involving suppression of the PI3K/Akt/mTOR axis. Thyroid Hormones (THs) are key endocrine regulators of skeletal muscle metabolism and adaptation, exerting context-dependent effects that promote either muscle atrophy or hypertrophy. Here, we identify Phosphoinositide-3-kinase interacting protein 1, Pik3ip1, as a critical regulator of TH-dependent muscle homeostasis. Transcriptomic profiling of skeletal muscle from muscle-specific D2 knockout (mD2KO) and TH Receptor knockout (TRKO) mice revealed a catabolic transcriptional program associated with increased Pik3ip1 expression. Consistently, Pik3ip1 expression negatively correlated with TH signaling *in vivo* and *in vitro*. Functional studies in C2C12 myotubes showed that Pik3ip1 overexpression suppresses Akt/mTOR signaling, indicating that its induction is sufficient to impair anabolic pathway activation. *In vivo*, Pik3ip1 expression was rapidly induced during denervation-induced muscle atrophy and remained persistently elevated in mD2KO and TRKO muscles, characterized by altered TH signaling. Sustained Pik3ip1 expression was associated with impaired activation of the Akt/mTOR pathway and enhanced muscle wasting. Conversely, TH treatment reduced Pik3ip1 levels, restored Akt/mTOR signaling, and promoted anabolic responses. Forced Pik3ip1 expression attenuated TH-induced Akt/mTOR phosphorylation, confirming its role as a mediator of TH-dependent anabolic regulation. Collectively, these findings identify Pik3ip1 as a key negative regulator of PI3K/Akt/mTOR signaling in skeletal muscle and establish the TH-Pik3ip1 axis as an important mechanism controlling muscle mass maintenance during atrophic conditions.

## Highlights

• Pik3ip1 is identified as a novel Thyroid Hormone-responsive inhibitor of PI3K/Akt/mTOR signaling in skeletal muscle.
• Impaired TH signaling causes sustained Pik3ip1 upregulation and exacerbates denervation-induced muscle atrophy.
• Pik3ip1 overexpression suppresses anabolic Akt/mTOR activation, linking TH deficiency to catabolic remodeling.
• The TH-Pik3ip1 axis emerges as a critical mechanism controlling skeletal muscle adaptation during atrophic stress.

## Graphical abstract

**Figure:**
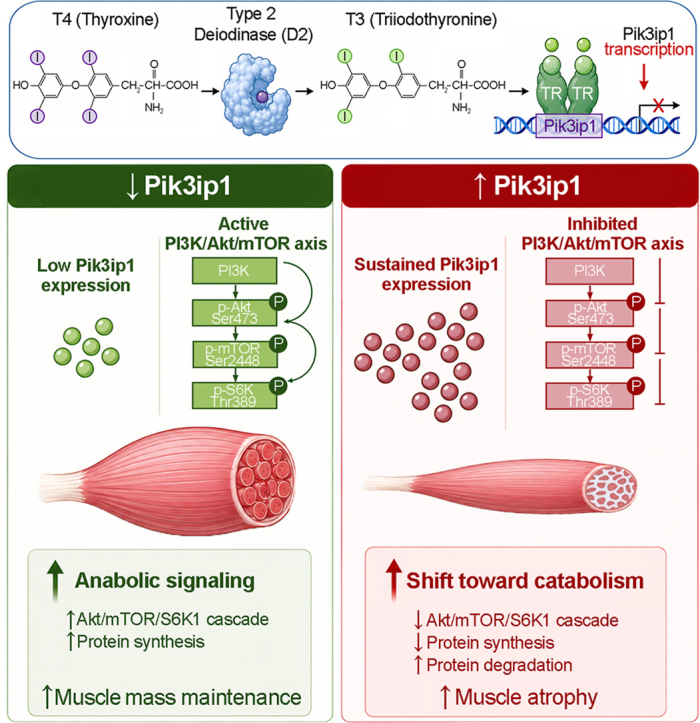

## 1 Introduction

Skeletal muscle is a highly plastic tissue that dynamically adapts to developmental, metabolic, and environmental stimuli through coordinated transcriptional and signaling networks. Among the endocrine regulators controlling skeletal muscle homeostasis, Thyroid Hormones (THs) play a central role in the regulation of muscle metabolism, mitochondrial activity, contractile function, and regenerative capacity [[1], [2], [3], [4], [5], [6], [7]]. Through Thyroid Hormone Receptors (TRs), TH signaling modulates broad transcriptional programs involved in energy expenditure, substrate utilization, and maintenance of proteostatic balance [[8], [9], [10], [11]]. Accordingly, disruption of TH signaling has been associated with impaired oxidative metabolism, defective regeneration, and increased susceptibility to muscle wasting [[12], [13], [14]].

Intracellular TH availability is tightly controlled by deiodinases, which locally regulate triiodothyronine (T3) production independently of circulating hormone levels. In skeletal muscle, type 2 deiodinase (D2) converts thyroxine (T4) into biologically active T3, thereby amplifying local TH signaling [[15], [16], [17], [18], [19]]. Previous studies demonstrated that D2-mediated T3 production is required for appropriate muscle adaptation under both physiological and pathological conditions, including regeneration and metabolic stress [9,12,20,21].

Denervation-induced muscle atrophy is characterized by profound transcriptional and metabolic reprogramming associated with suppression of anabolic signaling and activation of proteolytic pathways [[22], [23], [24], [25]]. A central event in this process is the dysregulation of the PI3K/Akt/mTOR pathway, which controls protein synthesis, muscle growth, and cellular adaptation to stress. Impaired Akt signaling promotes FOXO-dependent transcriptional programs that drive protein degradation and progressive muscle wasting [[26], [27], [28]]. Although TH signaling has been implicated in skeletal muscle trophism and metabolism, the mechanisms through which TH-dependent pathways influence anabolic adaptation during denervation remain poorly understood.

Here, through transcriptomic profiling of skeletal muscle from control, muscle-specific D2 knockout (mD2KO), and TRs knockout (TRKO) mice, we identified Phosphoinositide-3-kinase interacting protein 1 (Pik3ip1) as a consistently upregulated TH-responsive gene under conditions of impaired TH signaling. Pik3ip1 is a transmembrane inhibitor of PI3K signaling known to negatively regulate Akt pathway activation [29,30]. While Pik3ip1 has been extensively investigated in immune and cancer biology [31,32], only limited evidence is available regarding its role in striated muscle. Previous studies have implicated Pik3ip1 in the regulation of muscle cell differentiation through modulation of PI3K/Akt signaling and have identified its expression among genes associated with androgen-dependent skeletal muscle atrophy [33,34]. In cardiac muscle, Pik3ip1 has also been reported to negatively regulate hypertrophic growth by restraining PI3K signaling [30]. However, its involvement in TH-dependent regulation of muscle homeostasis and neurogenic muscle atrophy has not been explored.

Our findings identify Pik3ip1 as part of a conserved transcriptional program under TH signaling control acting as trigger of catabolic remodeling in skeletal muscle. We further show that Pik3ip1 expression is dynamically regulated during denervation while it remains persistently elevated in TH-deficient muscles, causing altered Akt/mTOR pathway activation and exacerbated atrophic remodeling. Together, these data support a model in which TH signaling contributes to the adaptive response to denervation, at least in part, through negative regulation of Pik3ip1 and preservation of anabolic signaling pathways.

## 2 Materials and methods

### 2.1 Cell culture and reagents

Murine C2C12 myoblasts (ATCC® CRL-1772™, RRID: CVCL_0188) were purchased from the American Type Culture Collection (ATCC, Manassas, VA, USA). Cells were authenticated by Short Tandem Repeat (STR) profiling, confirming complete correspondence with the reference ATCC C2C12 profile. Moreover, according to the International Cell Line Authentication Committee (ICLAC) database, the cell line has not been reported as misidentified or cross-contaminated. Mycoplasma testing was routinely performed before and during the experimental period using PCR-based detection methods, and all samples were confirmed negative. C2C12 cells were grown in Dulbecco's Modified Eagle Medium (DMEM; HiMedia Leading BioSciences Company, Mumbai, Maharashtra, India; Cat. No. AL007) supplemented with 10% Fetal Bovine Serum (FBS; HiMedia Leading BioSciences Company, Mumbai, Maharashtra, India; Cat. No. RM10432), 2.0 mM l-Glutamine (Gibco, Thermo Fisher Scientific, Waltham, MA, USA; Cat. No. 25030024), and 50 IU/mL Penicillin/Streptomycin (Gibco, Thermo Fisher Scientific, Waltham, MA, USA; Cat. No. 15070063) and maintained at 37 °C in a humidified incubator with 5% CO2. For induction of myogenic differentiation, C2C12 myoblasts at 60–70% confluence were shifted to differentiation medium consisting of DMEM supplemented with 2% Horse Serum (Sigma–Aldrich St. Louis, MI, USA, Catalog number: H1138), 10.0 μg/mL Insulin (Sigma–Aldrich St. Louis, MI, USA, Catalog number: I2643), and 5.0 μg/mL Transferrin (Sigma–Aldrich St. Louis, MI, USA, Catalog number: T8158). For TH treatment, differentiation medium was supplemented with 30.0 nM Triiodothyronine (T3, 3,3′,5-Triiodo-l-thyronine sodium salt, Sigma–Aldrich St. Louis, MI, USA, Catalog number: T6397) and thyroxine (T4, l-Thyroxine sodium salt pentahydrate, Sigma–Aldrich St. Louis, MI, USA, Catalog number: T2501), throughout the entire differentiation period, as indicated.

For transient transfection assays, C2C12 cells were transfected either with a Pik3ip1 overexpression construct (pEF-PIK3IP1 plasmid Cat. No. #49214, Addgene, Watertown, MA, USA) or with a PIK3IP1 shRNA plasmid (Santa Cruz Biotechnology, Dallas, TX, USA; Cat. No. sc-152262-sh) using Lipofectamine 2000 reagent (Invitrogen™, Carlsbad, CA, USA; Cat. No. 11668019), according to the manufacturer's instructions. Cells transfected with the corresponding empty vector or with a non-targeting control shRNA plasmid (Control shRNA Plasmid-A, Santa Cruz Biotechnology, Dallas, TX, USA; Cat. No. sc-108060) were used as negative controls, respectively.

### 2.2 Mouse models

All experimental procedures involving animals were performed in compliance with national and European regulations and received approval from the Italian Ministry of Health and the local Institutional Animal Care and Use Committee (IACUC, Project number 354/2019-PR, Protocol number D5A89.38, Project number 1/2024-PR, Protocol number D5A89.73 and Project number 757/2025-PR, Protocol number D5A89.105). Animals were maintained under specific pathogen-free conditions in a controlled environment at 22 ± 2 °C, with regulated humidity, a 12 h light/dark cycle, and unrestricted access to chow and water. Genotypes were confirmed by Polymerase Chain Reaction (PCR) analysis on genomic DNA isolated from tail biopsies using allele-specific primer sets. Muscle-specific *Dio2*-deficient mice (mD2KO) were obtained by breeding D2Lox/Lox mice with transgenic mice expressing Cre recombinase under the Myosin Light Chain (MLC) promoter (STOCK Myl1tm1(cre)Sjb/J; Strain #024713; RRID: IMSR: JAX:024713) [35]. Both parental lines were maintained on a C57BL/6J background, and experimental mD2KO mice and littermate controls were generated from the same breeding colony. D2Lox/Lox littermates lacking the Cre transgene served as control animals (CTR). No genome-wide SNP analysis was performed to further assess the genetic background. To investigate the role of Thyroid Hormone Receptor signaling, TRα/TRβ double knockout mice (TRαKO/TRβKO) were also included where indicated [36,37]. These animals harbor targeted deletions of the *Thra* and *Thrb* loci and were maintained on a C57BL/6J background. No inducible Tamoxifen-dependent Cre systems were employed in this study.

For Western Blot and RNA-sequencing studies, gastrocnemius muscles were obtained from 8-week-old male mice to limit variability arising from sex-related hormonal differences. Validation analyses were subsequently conducted in separate cohorts comprising male and female animals.

Tissues collected for molecular analyses were immediately snap-frozen in liquid nitrogen or on dry ice. For molecular analyses, whole gastrocnemius muscles were collected and immediately snap-frozen on dry ice. Frozen muscles were subsequently pulverized under liquid nitrogen using a pre-chilled mortar and pestle to obtain a homogeneous tissue powder. Aliquots of the resulting powder were then used for RNA or protein extraction, as appropriate.

For histological and immunofluorescence evaluations, tibialis anterior muscles were embedded in Tragacanth gum (Sigma–Aldrich, St. Louis, MO, USA; Cat. No. G1128) on cork supports, frozen in pre-cooled 2-methylbutane (Sigma–Aldrich, St. Louis, MO, USA; Cat. No. 277258) for 1 min, then transferred to liquid nitrogen and maintained at −80 °C until analysis [38].

For THs treatment experiments, 12-week-old male and female mice received triiodothyronine (T3; 3,3′,5-triiodo-l-thyronine sodium salt, Sigma–Aldrich, St. Louis, MO, USA; Cat. No. T6397) and thyroxine (T4; l-thyroxine sodium salt pentahydrate, Sigma–Aldrich, St. Louis, MO, USA; Cat. No. T2501) through the drinking water for 15 days. T3 and T4 were administered at final concentrations of 0.24 μg/mL and 0.032 μg/mL, respectively, corresponding to estimated daily doses of approximately 0.038–0.058 mg/kg/day for T3 and 0.0051–0.0077 mg/kg/day for T4. Experimental hypothyroidism was induced by administration of Methimazole (MMI, 0.1%, (Sigma–Aldrich, St. Louis, MO, USA; Cat. No. M8506) and potassium perchlorate (KClO_4_, 1%) in the drinking water for 6 weeks. Animals were sacrificed and skeletal muscles were collected immediately at the end of the treatment period. Systemic thyroid status was confirmed by serum TH measurements, including circulating T3, T4 and TSH levels, collected at the time of sacrifice.

### 2.3 Serum thyroid hormone, insulin and insulin-like growth factor 1 measurements

Serum T3 and T4 measurements were determined as previously described [[39], [40], [41]]. Whole blood was collected in anticoagulant-free tubes and allowed to clot at room temperature prior to processing. Serum was separated by centrifugation at 3.000 r.p.m. for 15 min at 4 °C, aliquoted and stored at −20 °C until analysis. Serum total T3 and T4 concentrations were determined by chemiluminescent immunoassay using the ADVIA Centaur XP platform (Siemens Healthcare Diagnostics, Camberley, UK), following the manufacturer's recommendations. Circulating Insulin and Insulin-like Growth Factor 1 (IGF1) concentrations were measured in mouse serum collected at the indicated time-points. Serum Insulin levels were determined using the Mouse Insulin ELISA Kit (Gibco, Thermo Fisher Scientific, Waltham, MA, USA; Cat. No. EMINS), while serum IGF1 concentrations were quantified using the Mouse IGF-1 ELISA Kit (Gibco, Thermo Fisher Scientific, Waltham, MA, USA; Cat. No. EMIGF1), according to the manufacturer's instructions.

### 2.4 Intramuscular thyroid hormone measurements

T3 and T4 levels in frozen muscle samples were determined by Liquid Chromatography-Tandem Mass spectrometry (LC-MS/MS) as previously described [42]. Iodothyronines were separated by reversed-phase chromatography on a Waters BEH C18 column (130 Å, 1.7 μm, 2.1 × 50 mm) using an Acquity UPLC-Xevo TQ-S tandem mass spectrometer (Waters, Milford, MA, USA). Mobile phases consisted of acetonitrile/H_2_O/acetic acid (5:95:0.1, v/v/v; phase A) and acetonitrile/H_2_O/acetic acid (95:5:0.1, v/v/v; phase B). An increasing gradient from 10% to 40% phase B was applied, with a total run time of 12 min. Analytes were quantified by Multiple Reaction Monitoring (MRM) using isotopically labeled internal standards. Data acquisition and processing were performed with MassLynx v4.1 and TargetLynx software (Waters).

### 2.5 *In vivo* skeletal muscle denervation

Sciatic nerve denervation was performed in adult mice under general anesthesia induced with 100 mg/kg Ketamine. Briefly, the right hindlimb was shaved and disinfected, and a small skin incision was made at the mid-thigh level to expose the sciatic nerve. A 5–7 mm segment of the sciatic nerve was carefully isolated and excised to prevent reinnervation. The contralateral limb was used as an internal control (Innervated muscle, as specified). Following surgery, the incision was closed using surgical sutures or wound clips, and animals were allowed to recover under standard housing conditions with free access to food and water. Mice were sacrificed at the indicated time points after denervation, and skeletal muscles were rapidly dissected, weighed, snap-frozen in liquid nitrogen, and stored at −80 °C until further analyses.

### 2.6 Immunofluorescence analysis of differentiated C2C12 cells

Differentiated C2C12 myotubes cultured on glass coverslips were fixed for 30 min in a cold solution containing 70% Methanol/30% Acetone. Cells were then permeabilized with 0.2% Triton X-100 in PBS for 15 min and blocked for 1 h at Room Temperature (RT) in Phosphate Buffered Saline (PBS) containing 2% Bovine Serum Albumin (BSA). Samples were incubated overnight at 4 °C with rabbit anti-PIK3IP1 polyclonal antibody (anti-PIK3IP1 polyclonal antibody Cat. No. PA5-100172, diluted 1:100 in blocking solution). Following PBS washes, filamentous actin was stained with Phalloidin (Phalloidin Labeling Probe Alexa Fluor™ 488 Invitrogen™, Carlsbad, CA, USA; Cat. No. A12379) diluted 1:100 from a 0.5 mg/mL stock solution at a final concentration of 5 μg/mL. Cells were subsequently incubated with the appropriate fluorescent secondary antibody (anti-rabbit IgG H + L Highly Cross-Adsorbed Secondary Antibody, Alexa Fluor 594, Invitrogen™, Carlsbad, CA, USA; Cat. No. A-21207, diluted 1:300 in blocking solution), washed with PBS, and counterstained with DAPI (4′,6-diamidino-2-phenylindole dihydrochloride; Invitrogen™, Carlsbad, CA, USA; Cat. No. D1306) to visualize nuclei. Coverslips were mounted using 80% glycerol. Images were acquired using a Leica DMi8 fluorescence microscope under identical exposure settings and analyzed with ImageJ Fiji software (NIH Image, Bethesda, MD, USA).

### 2.7 Histological and Immunohistochemical analyses of skeletal muscle sections

For Hematoxylin and Eosin (H/E) staining, muscle cryosections were fixed in 4% ParaFormAldehyde (PFA) for 15 min at RT and processed according to standard histological procedures. Myofiber Cross-Sectional Area (CSA) distribution was quantified using ImageJ software. Up to five images per muscle were acquired from anatomically comparable regions, and approximately 500 myofibers were analyzed for each sample. Image acquisition was performed using a Leica DMi8 microscope equipped with Leica Application Suite software (Leica Microsystems GmbH, Wetzlar, Germany). For immunofluorescence analyses, sections were fixed in 4% PFA and subjected to antigen retrieval in 0.1 M Sodium Citrate buffer (pH 6.0) by microwave heating. Tissue sections were then blocked in PBS containing 3% normal goat serum and 0.3% Triton X-100 prior to overnight incubation at 4 °C with the Pik3ip1 primary antibody (anti-PIK3IP1 polyclonal antibody Cat. No. PA5-100172, diluted 1:1000 in Tris-Buffered Saline (TBS) containing 5% Bovine Serum Albumin (BSA) and 0.2% Tween-20). After washing, sections were incubated for 1 h at RT with the appropriate Alexa Fluor cross-adsorbed secondary antibody (anti-rabbit IgG H + L Alexa Fluor 488, Invitrogen™, Carlsbad, CA, USA; Cat. No. A-11070, diluted 1:300 in blocking solution). Wheat Germ Agglutinin (WGA, Alexa Fluor 555 conjugate, Invitrogen™, Carlsbad, CA, USA; Cat. No. W32464) staining was subsequently performed at a final concentration of 10 μg/mL for 15 min at RT to delineate myofiber boundaries. Nuclei were counterstained with DAPI (4′,6-diamidino-2-phenylindole dihydrochloride; Invitrogen™, Carlsbad, CA, USA; Cat. No. D1306), and sections were mounted in 80% glycerol. Fluorescence images were acquired using a Leica DMi8 fluorescence microscope and analyzed with ImageJ Fiji software (NIH Image, Bethesda, MD, USA).

### 2.8 RNA-seq library preparation and differential gene expression (DEG) analysis

RNA extracted was quantified using a Qubit 4.0 fluorometric assay (Thermo Fisher Scientific, Waltham, MA, USA) as previously described in Colicchio et al., 2015 [43]. For each sample, 125.0 ng of total RNA were used for library preparation. Libraries were generated using the NEGEDIA Digital mRNA-seq research grade sequencing service v2.0 (Next Generation Diagnostic s.r.l.), which included library preparation, quality control, and sequencing. Sequencing was carried out on an Illumina NovaSeq X platform using a single-end 100-cycle run configuration. Raw Base Call (BCL) files were converted into FASTQ format using BCL Convert. Sequencing reads underwent quality control and adapter trimming with BBDuk prior to alignment against the mouse reference genome (mm10) using STAR v2.6.0a. Gene-level read counts were obtained using HTSeq-count v0.9.1, and raw count matrices were exported as tab-delimited text files. Normalization and downstream differential expression analyses were performed using the proprietary NEGEDIA DEGs analysis pipeline (v2.0; Next Generation Diagnostic s.r.l.) [[44], [45], [46]]. Statistical significance was determined using adjusted p-values (pAdj ≤0.05) following Benjamini-Hochberg False Discovery Rate (FDR) correction for multiple testing. An initial threshold of |Log2FC| ≥ 0.58 (corresponding to an approximately 1.5-fold change) was applied to identify biologically relevant transcriptional changes [41]. To identify the most robust and biologically relevant transcriptional alterations shared between mD2KO and TRKO muscles, subsequent analyses were restricted to genes meeting a more stringent threshold of p-value ≤0.05 and |Log2FC| ≥ 1.0. The complete lists of Differentially Expressed Genes (DEGs) identified in the mD2KO *versus* CTR and TRKO *versus* CTR comparisons are provided in the Supplementary Materials.

### 2.9 Gene Ontology (GO) enrichment analysis

Gene Ontology (GO) enrichment analysis was independently performed on the significantly upregulated and downregulated gene clusters identified by RNA-seq, as previously reported in Torabinejad et al., 2025 [47]. Overrepresentation analysis was performed using Enrichr (https://maayanlab.cloud/Enrichr/) by interrogating the Gene Ontology (GO) Biological Process and MGI (Mouse Genome Informatics) Mammalian Phenotype gene set libraries. Enriched categories were ranked according to p-values and combined scores, and statistical significance was established at p ≤ 0.05 following multiple testing correction. To further assess pathway-level alterations, Gene Set Enrichment Analysis (GSEA) was conducted using the Molecular Signatures DataBase (MSigDB) Mouse Collection (https://www.gsea-msigdb.org/gsea/index.jsp). Enrichment analysis was performed on a pre-ranked gene list generated according to Log2 Fold Change values. GSEA results were represented through heatmaps, volcano plots, bubble plots, and functional clustering visualizations, as specified in the corresponding figures.

### 2.10 *In silico* promoter analysis for searching Transition Factor (TF) binding sites

The analysis of the Thyroid Hormone Receptor Binding Elements, also known as Thyroid Hormone Response Elements (TREs, AC M00239, ID V$T3R_01), in the murine Pik3ip1 promoter region was performed as previously described in Nappi et al., 2023 [48]. Briefly, putative TRE consensus sequences located in the upstream regulatory region of the murine Pik3ip1 promoter were identified using TFBIND (https://tfbind.hgc.jp, accessed on 21 April 2026). Only binding sites with matrix similarity scores ≥0.70 (maximum score = 1.00) were retained for further analysis. The genomic positions of all predicted TREs are summarized in Table 1. Sequence logos were generated using WebLogo (https://weblogo.threeplusone.com/create.cgi).

**Table 1:** Consensus sequence analysis of the putative motifs for Thyroid Hormone Receptor (AC M00239, ID V$T3R_01) within the Pik3ip1 promoter region.

Phosphoinositide-3-kinase interacting protein 1 (Pik3ip1)					
AC	ID	Score	Localization	Strand	Signal sequence
M00239	V$T3R_01	0.780617	419	+	CAATGAAGTCCCAGTA
M00239	V$T3R_01	0.832482	581	–	AAGGATGACCTTGAAC
M00239	V$T3R_01	0.734999	595	–	ACTTCTGACCCTCCTG
M00239	V$T3R_01	0.731781	739	+	ATCTGAGCTCAAATCT
M00239	V$T3R_01	0.850842	867	+	CTTAAAGGTCACACTT
M00239	V$T3R_01	0.749006	973	–	TGATCTGACCCTACAC
M00239	V$T3R_01	0.734242	1171	–	ACAGATGGCCTTATGC
M00239	V$T3R_01	0.805792	1292	+	GTTTGGGGACATGGTT
M00239	V$T3R_01	0.769638	1394	–	GCCTATAACCCCAGTT

### 2.11 Quantitative real-time PCR (qRT-PCR)

Total RNA was isolated from skeletal muscle tissues and cultured cells using TRIzol reagent (Life Technologies Ltd., Carlsbad, CA, USA; Cat. No. 15596018) according to the manufacturer's recommendations. To remove residual genomic DNA contamination, RNA samples were subjected to DNase treatment. RNA quantity and purity were assessed by spectrophotometric analysis. Complementary DNA (cDNA) synthesis was performed starting from 2.0 μg of total RNA using SuperScript™ VILO™ Master Mix (Life Technologies Ltd., Carlsbad, CA, USA; Cat. No. 11755-500) following the manufacturer's instructions [49].

Quantitative Real-Time PCR (qRT-PCR) reactions were carried out using SYBR Green Supermix (Bio-Rad, Hercules, CA, USA; Cat. No. 1708882) on a CFX Connect Real-Time PCR Detection System (Bio-Rad, Hercules, CA, USA; Cat. No. 1855201). PCR amplification conditions consisted of an initial denaturation step at 95 °C for 10 min, followed by 40 amplification cycles at 95 °C for 15 s and 60 °C for 1 min. Melting curve analysis was included at the end of each run to verify amplification specificity. All samples were analyzed in technical duplicates.

Relative transcript levels were normalized against the endogenous reference gene Cyclophilin-A (CypA), previously validated as stably expressed under all experimental conditions. Relative quantification values were calculated using the comparative Ct (2^−ΔΔCt^) method and reported as Fold Change (FC) relative to the calibrator group. Primers were designed to generate amplicons ranging from 100 to 300 bp and, whenever feasible, spanning exon/exon junctions. Primer sequences are listed in Table 2.

**Table 2:** List of oligonucleotides used for qRT-PCR.

Oligo	Name/Gene ID	Sense	Sequence
Cyclophilin A	*CypA*	Forward	CGCCACTGTCGCTTTTCG
		Reverse	AACTTTGTCTGCAAACAGCTC
CyA amplicon length	120 bp		
https://www.ncbi.nlm.nih.gov/gene/268373			
Gapdh	*Gapdh*	Forward	ACCACAGTCCATGCCATCAC
		Reverse	TCCACCACCCTGTTGCTGTA
Gapdh amplicon length	452 bp		
https://www.ncbi.nlm.nih.gov/datasets/gene/14433/			
Myogenin	*Myogenin*	Forward	TTGCTCAGCTCCCTCAACCAGGA
		Reverse	TGCAGATTGTGGGCGTCTGTAGG
Myogenin amplicon length	194 bp		
https://www.ncbi.nlm.nih.gov/datasets/gene/17928/			
Pik3ip1	*Pik3ip1*	Forward	AACCACAACTACTGCCGGAA
		Reverse	CACCTGTGCCTCTTTGTCAC
Pik3ip1 amplicon length	198 bp		
https://www.ncbi.nlm.nih.gov/gene/216505			

### 2.12 Western Blot (WB) analysis

Protein extracts were prepared from skeletal muscle tissues or C2C12 cultured cells using ice-cold Protein Lysis Buffer supplemented with protease and phosphatase inhibitor cocktails, as reported elsewhere [50]. Samples were homogenized on ice and centrifuged at 3.000 r.p.m. for 10 min at 4 °C to remove insoluble debris. Total protein concentration was determined using the Bio-Rad Protein Assay Dye Reagent (Bio-Rad, Hercules, CA, USA; Cat. No. 5000006) according to the manufacturer's instructions. Equal amounts of protein (20/30 μg) were mixed with Laemmli Sample Buffer containing β-mercaptoethanol, denatured at 99 °C for 7 min, and separated by Sodium Dodecyl Sulfate-PolyAcrylamide Gel Electrophoresis (SDS-PAGE) on polyacrylamide gels of appropriate percentage depending on target protein molecular weight. Proteins were subsequently transferred onto PolyVinylidene DiFluoride (PVDF) membranes using wet transfer systems. Membranes were blocked for 1 h at RT in Tris-Buffered Saline (TBS) containing 0.2% Tween-20 (TBS-Tween) supplemented with 5% Bovine Serum Albumin (BSA, Sigma–Aldrich, St. Louis, MO, USA; Cat. No. A9647-100G). Membranes were then incubated overnight at 4 °C with the indicated primary antibodies (listed in Table 3) diluted in blocking solution. Following washing steps in TBS-Tween, membranes were incubated for 1 h at RT with species-appropriate HorseRadish Peroxidase (HRP)-conjugated secondary antibodies (Goat Anti-Rabbit IgG-HRP Conjugate, blotting-grade horseradish peroxidase secondary antibody conjugate, Bio-Rad, Hercules, CA, USA; Cat. No. 1706515 or Goat Anti-Mouse IgG-HRP conjugate, blotting-grade horseradish peroxidase secondary antibody conjugate, Bio-Rad, Hercules, CA, USA; Cat. No. 1706516, diluted 1:3000 in blocking solution). Immunoreactive bands were detected using Enhanced Chemi-Luminescence (ECL) substrate and acquired with a digital imaging system. Densitometric analyses were performed using ImageJ software. Protein expression levels were normalized to housekeeping proteins or to the corresponding total protein levels for phosphorylated targets, as indicated in the corresponding figure legends.

**Table 3:** List of antibodies used for Western Blot.

Antibody	WB dilution	Catalog no. and provider
Akt	1:1000	Cell signaling Technology #9272
https://www.cellsignal.com/products/primary-antibodies/akt-antibody/9272		
Phospho-Akt (Ser473)	1:1000	Cell signaling Technology #9271
https://www.cellsignal.com/products/primary-antibodies/phospho-akt-ser473-antibody/9271		
mTOR	1:1000	Cell signaling Technology #2983
https://www.cellsignal.com/products/primary-antibodies/mtor-7c10-rabbit-monoclonal-antibody/2983		
Phospho-mTOR (Ser2448)	1:1000	Cell signaling Technology #5536
https://www.cellsignal.com/products/primary-antibodies/phospho-mtor-ser2448-d9c2-rabbit-monoclonal-antibody/5536		
S6K1	1:1000	Abcam ab131440
https://www.abcam.com/en-us/products/primary-antibodies/s6k1-antibody-ab131440		
Phospho-p70 S6K (Thr389)	1:1000	Cell signaling Technology #9205
https://www.cellsignal.com/products/primary-antibodies/phospho-p70-s6-kinase-thr389-antibody/9205		
Pik3ip1	1:1000	Invitrogen #PA5-100172
https://www.thermofisher.com/antibody/product/PIK3IP1-Antibody-Polyclonal/PA5-100172		
Alpha-Tubulin	1:5000	Cell signaling Technology #2144
https://www.cellsignal.com/products/primary-antibodies/alpha-tubulin-antibody/2144		

### 2.13 Statistical analysis

Statistical analyses were performed using GraphPad Prism (GraphPad Software Inc., La Jolla, CA, USA). Comparisons between two experimental groups were carried out using an unpaired two-tailed Student's t-test, as appropriate. For analyses involving multiple groups, one-way ANOVA followed by Tukey's post hoc multiple comparisons test was employed. Statistical significance was established at p < 0.05 and denoted as follows: p < 0.05 (∗), p < 0.01 (∗∗), p < 0.001 (∗∗∗) and p < 0.0001 (∗∗∗∗). Data are presented as mean ± Standard Deviation (SD).

## 3 Results

### 3.1 Impaired thyroid hormone signaling is associated with Pik3ip1 upregulation within a catabolic transcriptional program in skeletal muscle

**Figure 1:**
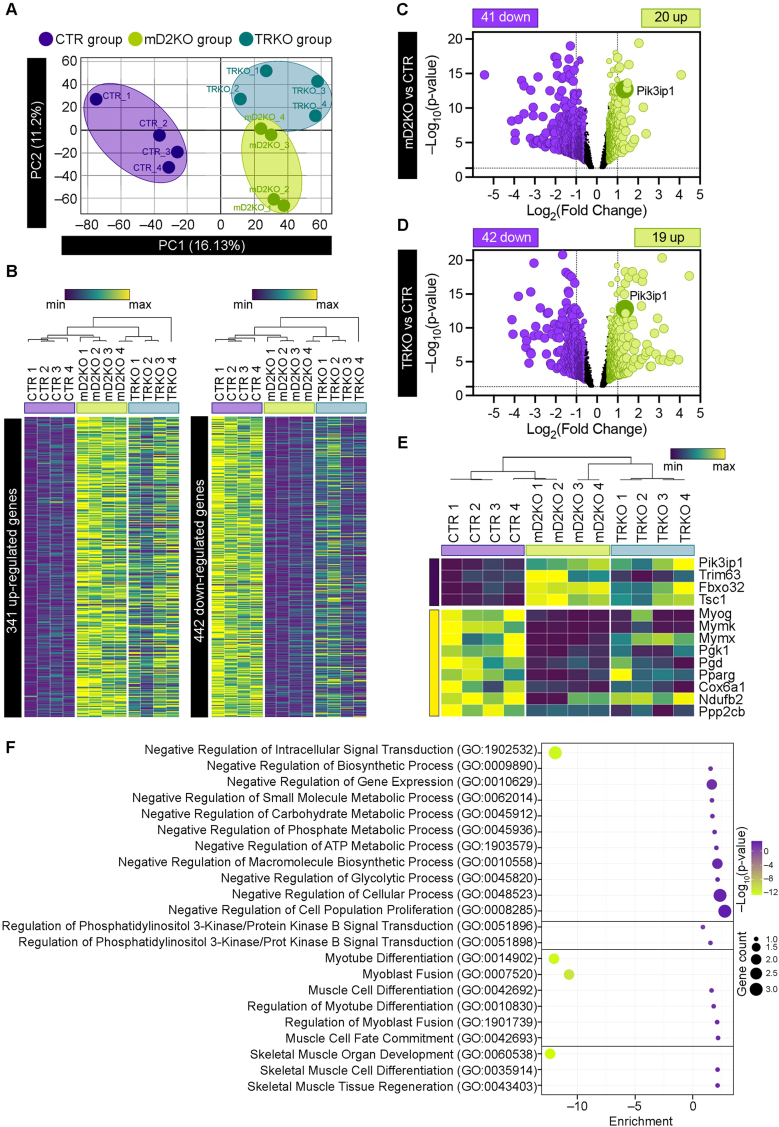
**Transcriptomic characterization identifies Pik3ip1 as a Thyroid Hormone-dependent repressor of myogenic differentiation pathways. (A)**Principal Component Analysis (PCA) of RNA-sequencing datasets derived from skeletal muscle samples of CTR, muscle-specific *Dio2* knockout (mD2KO), and TRKO mice. **(B)** Heatmaps showing hierarchical clustering of Differentially Expressed Genes (DEGs) in mD2KO and TRKO muscles compared with CTR. DEGs were identified using p < 0.05, |–0.58 ≤ Log2 fold change ≥0.58| thresholds. Color scale represents normalized expression levels from minimum (purple) to maximum (yellow). **(C**–**D)** Volcano plots showing significantly deregulated transcripts in mD2KO *versus* CTR **(C)** and TRKO *versus* CTR **(D)** muscles. DEGs were identified using p < 0.05, |–1.0 ≤ Log2 fold change ≥1.0| thresholds. **(E)** Heatmap of selected genes associated with skeletal muscle differentiation and fusion pathways across CTR, mD2KO, and TRKO groups. **(F)**Gene Ontology (GO) enrichment analysis of deregulated transcripts in mD2KO/TRKO muscles. Downregulated pathways were predominantly associated with skeletal muscle differentiation, myoblast fusion, muscle cell fate commitment, and tissue regeneration, whereas upregulated pathways included negative regulation of intracellular signal transduction, biosynthetic processes, carbohydrate metabolism, and PI3K/AKT signaling. Dot size indicates gene count and color scale represents −Log10 (p-value).

To define transcriptional programs regulated by TH signaling in skeletal muscle, we performed RNA-seq on gastrocnemius muscle from control (CTR), muscle-specific D2 knockout (mD2KO), and global TR knockout (TRKO) mice. Principal Component Analysis (PCA) score plots showed a clear segregation of knockout samples from CTR along the main axes of variance (Figure 1A). mD2KO and TRKO samples clustered closer to each other than to CTR, consistent with a shared transcriptional response to impaired TH signaling, while their partial overlap suggests that D2-driven T3 availability and TR-mediated transcription shape the hormonal response through partially distinct mechanisms. Differential expression analysis (p < 0.05, |–0.58 ≤ Log2 fold change ≥0.58|) further supported this separation, identifying a substantial number of significantly deregulated genes across the datasets (341 upregulated *versus* 442 downregulated genes, Figure 1B). To prioritize high-confidence and shared transcriptional changes, we applied a more stringent cutoff (p < 0.05, |–1.0 ≤ Log2 fold change ≥1.0|) and restricted the analysis to genes commonly deregulated in both mD2KO and TRKO conditions. This filtering reduced the dataset to a core transcriptional signature comprising about 20 upregulated and 40 downregulated genes (Figure 1C–D). Inspection of this gene set identified a restricted cluster of transcripts, with the first up-regulated gene Pik3ip1, a well-established mediator or marker of muscle atrophy [51,52], as well as regulators of myogenic differentiation (Myog, Mymk, Mymx) [53,54], alongside regulators of metabolism (Pgk1, Pgd) [55,56], mitochondrial function (Cox6a1, Ndufb2) [57,58], and signaling (Tsc1, Ppp2cb) [59,60] (Figure 1E and Fig. S1A). To define the functional context of this transcriptional program, we performed integrated EnrichR and Gene Set Enrichment Analysis (GSEA) (Figure 1F and Fig. S1B), both converging on two major biological themes: (i) muscle adaptation and response to inactivity, and (ii) negative regulation of anabolic metabolism and intracellular signaling, including PI3K/AKT-related pathways. The convergence of these transcriptional changes on atrophy-related pathways and inhibitory signaling nodes suggests that disruption of TH signaling promotes a coordinated catabolic program. Within this framework, Pik3ip1, a known negative regulator of PI3K signaling [32], emerged as a candidate upstream modulator of this response. Based on its consistent upregulation across both genetic models and its functional positioning within the identified pathways, we prioritized Pik3ip1 for further mechanistic investigation.

### 3.2 Pik3ip1 is a negatively regulated target of thyroid hormone signaling in skeletal muscle

**Figure 2:**
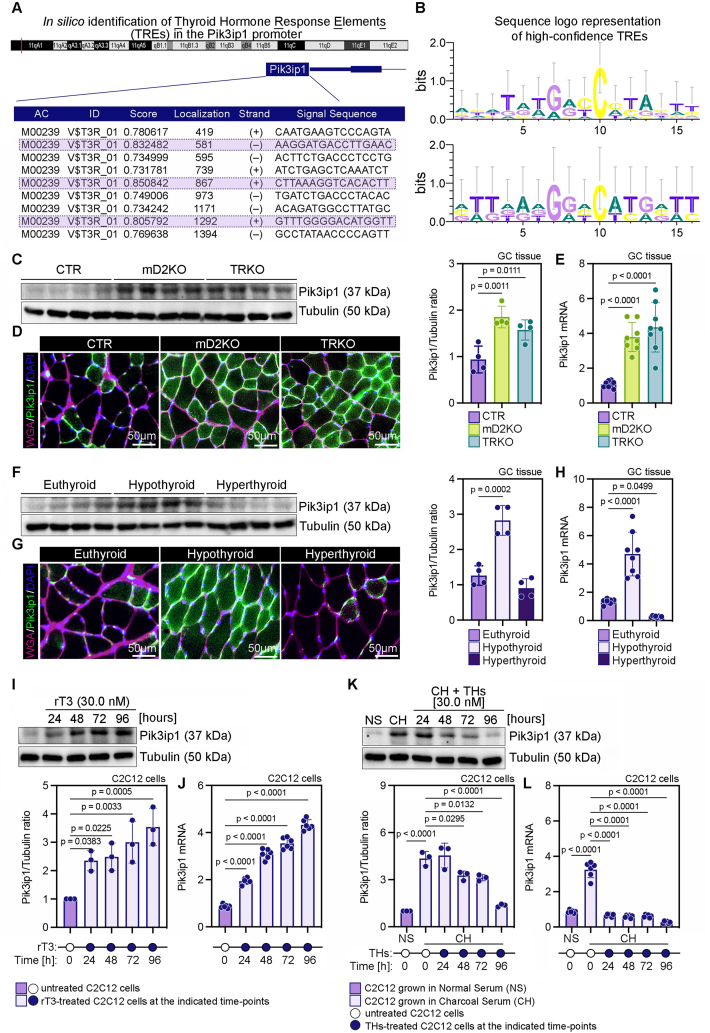
**Thyroid Hormone signaling negatively regulates Pik3ip1 expression in skeletal muscle. (A)** Schematic representation of predicted Thyroid Hormone Receptor Response Elements (TREs) identified within the murine Pik3ip1 promoter region. Putative TREs, their genomic localization, strand orientation, and prediction scores are shown. Highlighted sequences indicate the highest-confidence candidate binding motifs. **(B)** Sequence logos representing consensus TRE motifs identified within the Pik3ip1 regulatory regions. **(C)** Western Blot analysis of Pik3ip1 protein expression in skeletal muscle from CTR, mD2KO, and TRKO mice. Tubulin was used as loading control. Quantification of Pik3ip1/Tubulin ratio is shown on the right. **(D)** Representative immunofluorescence staining for Pik3ip1 (green) and WGA (magenta) in skeletal muscle sections from CTR, mD2KO, and TRKO mice. Nuclei were counterstained with DAPI (blue). Magnification 10x; scale bar 50 μm. **(E)** Relative Pik3ip1 mRNA expression in skeletal muscle from CTR, mD2KO, and TRKO mice. **(F)** Western Blot analysis of Pik3ip1 protein expression in skeletal muscle from euthyroid, hypothyroid, and hyperthyroid mice. Tubulin served as loading control. Quantification of Pik3ip1/Tubulin ratio is shown on the right. **(G)** Representative immunofluorescence staining for Pik3ip1 (green) and WGA (magenta) in skeletal muscle sections from euthyroid, hypothyroid, and hyperthyroid mice. Nuclei were stained with DAPI (blue). Magnification 10x; scale bar 50 μm. **(H)** Relative Pik3ip1 mRNA expression in skeletal muscle from euthyroid, hypothyroid, and hyperthyroid mice. **(I**–**J)** Western Blot and densitometric quantification of Pik3ip1 protein levels **(I)** and relative Pik3ip1 mRNA expression **(J)** in C2C12 myotubes treated with rT3 (30.0 nM) for the indicated times. **(K**–**L)** Western Blot and densitometric analysis of Pik3ip1 protein levels **(K)** and relative Pik3ip1 mRNA expression **(L)** in C2C12 cells cultured in CHarcoal-stripped medium (CH) and subsequently treated with Thyroid Hormones (THs: T3 + T4, 30.0 nM each) for the indicated times. NS, Normal Serum conditions. Data are presented as mean ± SD. Statistical significance was determined using one-way ANOVA followed by Tukey's multiple comparisons test.

To investigate whether Pik3ip1 is a transcriptional target of TH signaling, we first performed an *in silico* analysis on the murine promoter region, focusing on the 2000 bp upstream of the Transcription Start Site (TSS) (Figure 2A). *In silico* analysis of the Pik3ip1 promoter using the TRANSFAC matrix V$T3R_01 identified nine putative Thyroid Hormone Response Elements (TREs) with moderate matrix similarity scores (0.73–0.85) (Figure 2B, top panel). Filtering for higher-confidence matches (score ≥0.80) reduced the candidate sites to three (Figure 2A,B, bottom panel) and inspection of the underlying sequences revealed enrichment of consensus-related AGGTCA/GACCT-like half-sites, with 867-CTTAAAGGTCACACTT-882 and 581-AAGGATGACCTTGAAC-596 representing the most prominent candidates based on their higher similarity to the canonical motif. Although no canonical DR4-type TRE architecture (Direct Repeats separated by four nucleotides) was detected, the identified sites display sequence features consistent with consensus-related AGGTCA/GACCT-like half-sites, supporting the potential for non-canonical or context-dependent TR binding. Based on these observations, we hypothesized that Pik3ip1 expression may be responsive to TH signaling.

We then validated Pik3ip1 regulation in *in vivo* and in *vitro* models of altered TH signaling. Consistent with the transcriptomic analysis, Pik3ip1 mRNA and protein expression was significantly upregulated in skeletal muscle from both mD2KO and TRKO mice compared to CTR, indicating that both impaired local T3 production and loss of receptor-mediated signaling converge on Pik3ip1 induction (Figure 2C–E). To further assess TH dependency, we analyzed Pik3ip1 expression under systemic thyroid states. Pik3ip1 mRNA and protein levels were significantly increased in hypothyroid conditions and reduced under hyperthyroid conditions compared to euthyroid controls, demonstrating an inverse correlation with TH availability and reinforcing its classification as a negatively regulated TH target (Figure 2F–H).

To determine whether Pik3ip1 regulation is directly influenced by TH availability within the myogenic lineage, we analyzed its expression in C2C12 cells under conditions of altered TH signaling. The effectiveness of TH signaling alteration was confirmed by assessing the expression of the canonical T3-responsive protein Myogenin as a positive control, whereas GAPDH, used as a non-T3-responsive negative control (Fig. S2A and B). First, C2C12 cells were treated with reverse T3 (rT3), a known inhibitor of the D2-mediated intracellular T3 production, and Pik3ip1 levels were measured at defined time-points, 24, 48, 72, and 96 h. Under these conditions, Pik3ip1 expression showed a progressive increase over time, indicating that inhibition of intracellular T3 signaling is sufficient to induce its upregulation in C2C12 cells (Figure 2I,J). To further evaluate the contribution of extracellular TH, C2C12 cells were cultured in either Normal or Charcoal-stripped Serum, the latter depleted of endogenous THs, and treated or not with exogenous THs across the same time points. Under Charcoal-stripped conditions, Pik3ip1 expression was markedly elevated compared to normal conditions, and this effect was further modulated by THs supplementation (Figure 2K,L).

Collectively, these results identify Pik3ip1 as a negatively regulated TH-responsive gene in skeletal muscle, suggesting that alterations in Pik3ip1 expression may contribute to downstream modulation of anabolic signaling pathway.

### 3.3 Pik3ip1 overexpression dampens thyroid hormone-induced activation of the Akt/mTOR anabolic pathway in skeletal muscle cells

**Figure 3:**
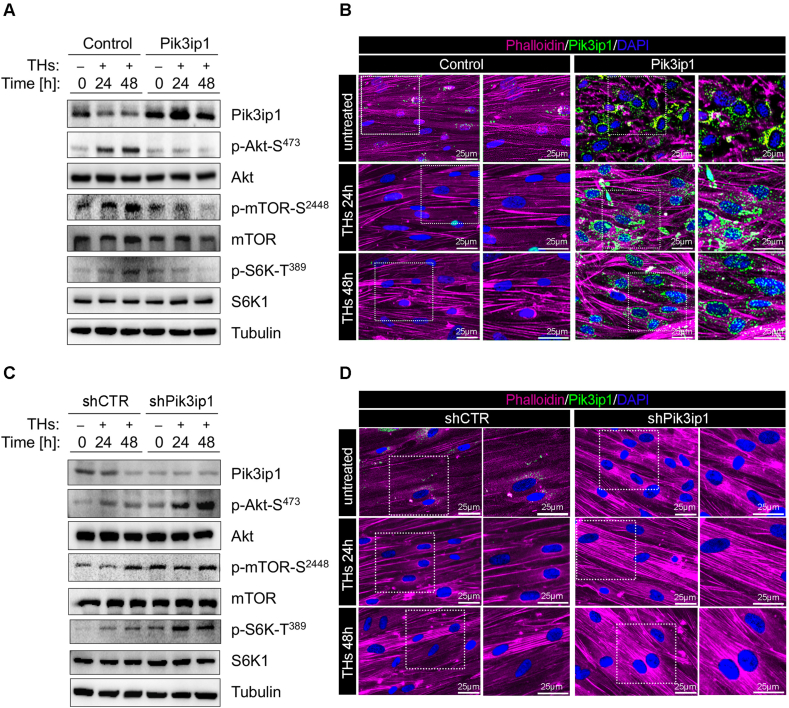
**Pik3ip1 regulates TH-mediated activation of the Akt/mTOR signaling pathway in skeletal muscle cells. (A)** Protein levels of Pik3ip1, phospho-Akt (Ser473), total Akt, phospho-mTOR (Ser2448), total mTOR, phospho-S6K1 (Thr389), and total S6K1 were evaluated by Western Blot in Control and Pik3ip1-overexpressing C2C12 cells treated with THs (T3 + T4, 30.0 nM each) for 24 or 48 h. Tubulin was used as loading control. **(B)** Representative immunofluorescence images of differentiated C2C12 myotubes transfected with either an empty vector (Control) or a Pik3ip1 expression plasmid and cultured in the absence or presence of THs (T3 + T4, 30.0 nM each) for 24 or 48 h. Cells were stained for Pik3ip1 (green), F-actin with Phalloidin (magenta), and nuclei with DAPI (blue). Dashed boxes indicate regions shown at higher magnification in the adjacent panels. Magnification 40x; scale bar 25 μm. **(C)** Protein levels of Pik3ip1, phospho-Akt (Ser473), total Akt, phospho-mTOR (Ser2448), total mTOR, phospho-S6K1 (Thr389), and total S6K1 were evaluated by Western Blot in differentiated C2C12 myotubes transfected with either a control shRNA plasmid (shCTR) or a Pik3ip1-targeting shRNA plasmid (shPik3ip1) and treated with THs (T3 + T4, 30.0 nM each) for 24 or 48 h. Tubulin was used as loading control. **(D)** Representative immunofluorescence images of differentiated C2C12 myotubes transfected with either a control shRNA plasmid (shCTR) or a Pik3ip1-targeting shRNA plasmid (shPik3ip1) and cultured in the absence or presence of THs (T3 + T4, 30.0 nM each) for 24 or 48 h. Cells were stained for Pik3ip1 (green), F-actin with Phalloidin (magenta), and nuclei with DAPI (blue). Dashed boxes indicate regions shown at higher magnification in the adjacent panels. Magnification 40x; scale bar 25 μm.

To investigate whether Pik3ip1 functionally mediates the effects of TH signaling on anabolic pathways in skeletal muscle cells, we next evaluated the impact of Pik3ip1 overexpression on Akt/mTOR signaling in C2C12 myotubes under TH-treated conditions (Figure 3A and Fig. S3A–D).

TH treatment increased Akt phosphorylation at Ser473 in control myotubes, with a more pronounced activation observed after 48 h, consistent with stimulation of anabolic signaling (Figure 3A and Fig. S3B). This effect was accompanied by increased phosphorylation of downstream mTORC1 targets, including mTOR at Ser2448 and S6K1 at Thr389 (Figure 3A and Fig. S3C–D). In contrast, Pik3ip1-overexpressing myotubes displayed a marked attenuation of TH-induced Akt activation, with substantially reduced p-Akt levels at both 24 and 48 h compared with control cells. Consistently, phosphorylation of mTOR and S6K1 was also reduced in Pik3ip1-overexpressing cells, despite comparable total Akt, mTOR, and S6K1 protein levels in all conditions. Notably, TH exposure did not restore anabolic signaling in the presence of elevated Pik3ip1 expression, suggesting that Pik3ip1 acts upstream of Akt as a negative regulator capable of counteracting TH-induced activation of the Akt/mTOR pathway. To further assess whether modulation of Akt/mTOR signaling was associated with TH-dependent morphological changes in differentiated myotubes, we performed immunofluorescence analyses using Phalloidin staining to visualize F-actin organization (Figure 3B and Fig. S3E. In control myotubes, TH treatment induced a progressive increase in myotube caliber and cytoskeletal organization, becoming more evident after 48 h of exposure. In contrast, Pik3ip1-overexpressing myotubes displayed a thinner and less organized morphology, consistent with impaired anabolic adaptation. Although TH treatment partially improved myotube architecture in Pik3ip1-overexpressing cells, the recovery remained incomplete compared with control myotubes, indicating that sustained Pik3ip1 expression limits TH-dependent structural remodeling. Morphometric analysis further confirmed this observation, revealing a progressive increase in myotube thickness following TH treatment in control cells, whereas Pik3ip1 overexpression significantly blunted this response (Figure 3B and Fig. S3E).

To determine whether Pik3ip1 is required to restrain TH-induced anabolic signaling, we next performed loss-of-function experiments in differentiated C2C12 myotubes using shRNA-mediated Pik3ip1 silencing (Figure 3C and Fig. S4). Efficient Pik3ip1 knockdown was confirmed by Western Blot analysis. Consistent with a negative regulatory role, Pik3ip1 silencing enhanced TH-induced activation of the Akt/mTOR pathway (Figure 3C and Fig. S4A). Compared with shCTR cells, shPik3ip1 myotubes displayed increased phosphorylation of Akt (Ser473), mTOR (Ser2448), and S6K1 (Thr389) following TH treatment (Figure 3C and Fig. S4B–D), whereas total protein levels remained unchanged. Notably, enhanced pathway activation was evident at both 24 and 48 h after TH exposure, indicating that endogenous Pik3ip1 normally constrains the magnitude of TH-induced anabolic signaling. Moreover, immunofluorescence analysis further supported these findings (Figure 3D and Fig. S4E). Efficient Pik3ip1 silencing markedly reduced the punctate cytoplasmic Pik3ip1 signal observed in shCTR myotubes. Under basal conditions, Pik3ip1 silencing promoted a hypertrophic phenotype, with shPik3ip1 myotubes displaying a larger caliber than shCTR myotubes. Upon TH stimulation, Pik3ip1-deficient myotubes underwent a more pronounced structural remodeling than shCTR cells, characterized by thicker myotubes and the formation of densely packed, longitudinally aligned actin bundles. These morphological differences became more evident after 48 h of TH exposure, indicating that depletion of endogenous Pik3ip1 enhances the cytoskeletal and trophic response to THs. These findings indicate that Pik3ip1 acts as a critical regulator of TH-dependent Akt/mTOR signaling in skeletal muscle cells. Gain- and loss-of-function approaches demonstrate that Pik3ip1 is sufficient to suppress, and required to restrain, TH-induced activation of the Akt/mTOR pathway. By modulating the magnitude of anabolic signaling, Pik3ip1 functionally controls the trophic response of differentiated myotubes to TH stimulation. These results also reinforce previous reports indicating that Pik3ip1-mediated inhibition of the PI3K/Akt/mTOR pathway contributes to the regulation of myogenic differentiation in proliferating myoblasts [33].

### 3.4 Impaired TH signaling sustains Pik3ip1 expression and prolongs catabolic remodeling during muscle denervation

**Figure 4:**
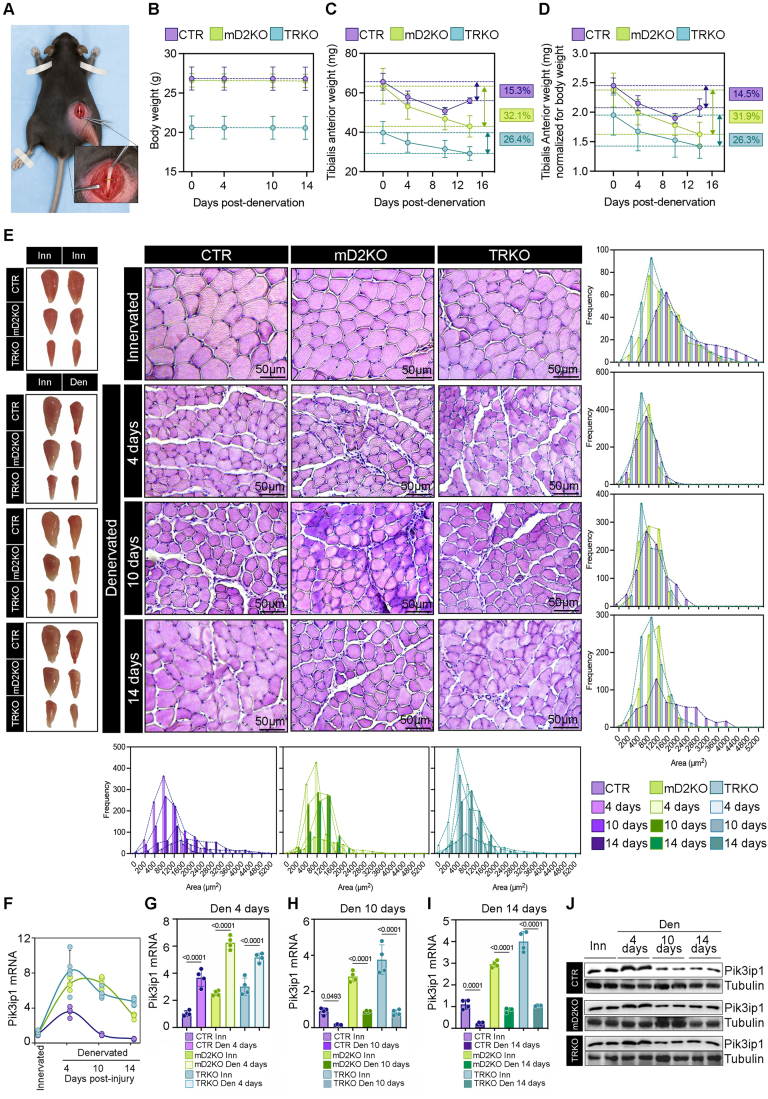
**Pik3ip1 expression is induced during denervation-induced skeletal muscle atrophy in mD2KO and TRKO muscles. (A)** Representative image of the sciatic nerve denervation procedure. Inset shows exposure and transection of the sciatic nerve. **(B)** Body weight measurements of CTR, mD2KO, and TRKO mice following denervation surgery over the indicated time course. **(C)**Tibialis Anterior (TA) muscle weight measured at the indicated time points after denervation. Percent reductions at day 14 are indicated. **(D)** TA muscle weight normalized to body weight following denervation. **(E)** Representative gross morphology and Hematoxylin and Eosin (H/E) staining of Innervated (Inn) and Denervated (Den) TA muscles from CTR, mD2KO, and TRKO mice collected at 4-, 10-, and 14-days post-denervation. Frequency distribution histograms of myofiber Cross-Sectional Area (CSA) are shown on the right (comparison inter genotypes over-time) and bottom panels (comparison intra genotypes over-time). Magnification 10x; scale bar 50 μm. **(F)** Relative Pik3ip1 mRNA expression in innervated and denervated TA muscles from CTR, mD2KO, and TRKO mice across the indicated time points. **(G**–**I)** The same dataset shown in panel F, displayed by individual time points to facilitate direct comparisons among genotypes at 4- **(G)**, 10- **(H)**, and 14- **(I)** days following denervation. **(J)** Western Blot analysis of Pik3ip1 protein expression in innervated and denervated TA muscles from CTR, mD2KO, and TRKO mice collected at the indicated time points. Tubulin was used as loading control. Data are presented as mean ± SD. Statistical significance was determined using one-way ANOVA followed by Tukey's multiple comparisons test.

Since Pik3ip1 negatively regulates Akt/mTOR signaling and was consistently induced under conditions of altered TH signaling, we next investigated its regulation during denervation-induced muscle remodeling. To this end, CTR, mD2KO, and TRKO mice were subjected to sciatic nerve transection and skeletal muscles were analyzed at 4-, 10-, and 14-days post-denervation (Figure 4A). Although no significant changes in body weight were observed throughout the experimental period within each genotype (Figure 4B), consistently with the development of muscle atrophy, denervation induced progressive loss of Tibialis Anterior (TA) muscle mass over time (Figure 4C). However, the extent of muscle loss differed between genotypes, with mD2KO and TRKO mice exhibiting a greater reduction in TA mass compared with CTR mice at the latest time point. Similar results were obtained after normalization of TA weight to body weight (Figure 4D), confirming that the enhanced reduction observed in mD2KO and TRKO muscles was not attributable to differences in total body mass. Consistently, histological and morphometric analyses further confirmed a greater reduction in myofiber size in mD2KO and TRKO mice (Figure 4E and Fig. S5A and B). Notably, whereas CTR muscles exhibited evidence of partial recovery/adaptive remodeling between days 10 and 14 post-denervation, this response was markedly impaired in mD2KO and TRKO muscles, indicating that defective TH signaling compromises the adaptive recovery process following denervation.

Interestingly, denervation did not affect circulating T3 or T4 levels in any genotype, indicating that the observed responses were not secondary to systemic alterations in thyroid status (Fig. S6A). In contrast, intramuscular TH levels displayed a dynamic profile (Fig. S6B). In CTR muscles (Fig. S6B and i), both T3 and T4 were markedly reduced at 4 days post-denervation, resulting in a significant decrease in the intramuscular T3/T4 ratio and suggesting an early local hypothyroid state. However, at 10- and 14-days post-denervation, intramuscular T3 levels and the T3/T4 ratio increased above basal values, consistent with a compensatory restoration of local TH signaling during the remodeling phase. This adaptive response was markedly altered in mD2KO and TRKO muscles (Figure S6B, ii and iii), supporting the notion that proper local TH signaling contributes to the temporal adaptation of skeletal muscle to denervation-induced remodeling.

Because insulin and IGF1 are major upstream activators of PI3K/Akt signaling, we next assessed whether systemic endocrine alterations could account for the differential anabolic responses observed among genotypes. However, circulating insulin and IGF1 levels did not significantly differ among CTR, mD2KO, and TRKO mice at any time point following denervation (Fig. S7A–C). These findings indicate that the altered Akt/mTOR signaling and the exacerbated atrophic phenotype observed in mutant muscles are unlikely to be explained by systemic endocrine differences and instead support a predominant contribution of local intramuscular mechanisms.

We next analyzed the temporal expression profile of Pik3ip1 during denervation-induced muscle remodeling. Interestingly, Pik3ip1 expression displayed a dynamic temporal profile during denervation. Pik3ip1 mRNA and protein levels were markedly induced at early stages following denervation and remained consistently higher in mD2KO and TRKO muscles relative to CTR muscles at all time points, despite a progressive decline during prolonged denervation (Figure 4F–J and Fig. S7D–F). Collectively, these findings suggest that impaired TH signaling disrupts the temporal adaptation to denervation-induced remodeling, resulting in enhanced muscle atrophy and sustained Pik3ip1 expression in mutant muscles.

### 3.5 Early Pik3ip1 induction associates with delayed anabolic adaptation during muscle denervation in TH-deficient mice

**Figure 5:**
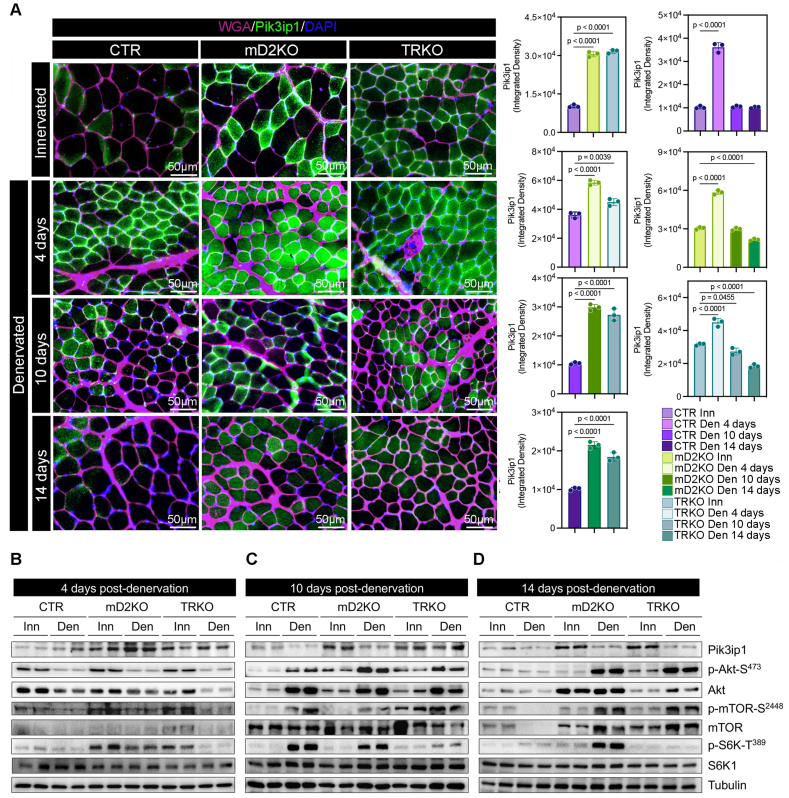
**Denervation-induced Pik3ip1 upregulation is associated with impaired AKT/mTOR signaling in mD2KO and TRKO skeletal muscle. (A)** Representative immunofluorescence staining of Pik3ip1 (green) in Innervated (Inn) and Denervated (Den) Tibialis Anterior (TA) muscles from CTR, mD2KO, and TRKO mice collected at 4-, 10-, and 14-days post-denervation. Wheat Germ Agglutinin (WGA, magenta) was used to outline myofiber membranes and nuclei were counterstained with DAPI (blue). Quantification of Pik3ip1 integrated fluorescence intensity is shown on the right. Magnification 10x; scale bar 50 μm. **(B**–**D)** Western Blot analyses of Pik3ip1 and components of the AKT/mTOR signaling pathway in Innervated (Inn) and Denervated (Den) TA muscles from CTR, mD2KO, and TRKO mice collected at 4- **(B)**, 10- **(C)**, and 14- **(D)** days following denervation. Protein levels of phospho-Akt (S473), total Akt, phospho-mTOR (S2448), total mTOR, phospho-S6K (T389), total S6K1, and Pik3ip1 were evaluated. Tubulin was used as loading control.

Given the dynamic regulation of Pik3ip1 expression observed during denervation, we next investigated its spatial distribution in skeletal muscle by immunofluorescence analysis across progressive stages of denervation in CTR, mD2KO, and TRKO muscles (Figure 5A). Consistent with the transcriptional and biochemical analyses, Pik3ip1 expression was low in innervated CTR muscles but markedly increased following denervation, particularly at early stages post-injury. In contrast, mD2KO and TRKO muscles displayed higher basal Pik3ip1 staining under innervated conditions and maintained stronger immunoreactivity throughout denervation compared with CTR muscles.

Temporal analysis revealed that Pik3ip1 progressively declined at later stages following denervation across all genotypes, consistent with the reduction observed by qPCR and Western Blot analyses. However, despite this decline, mD2KO and TRKO muscles retained higher Pik3ip1 levels compared with CTR muscles at all time points, indicating persistent dysregulation of Pik3ip1 expression under conditions of impaired TH signaling (Figure 5A). Notably, Pik3ip1 was predominantly enriched in denervated atrophic myofibers, further supporting the association between sustained Pik3ip1 expression and altered adaptive remodeling following denervation (Figure 5A).

To further define the molecular basis underlying this phenotype, we next examined the activation state of the Akt/mTOR anabolic pathway during progressive stages of denervation. Western Blot analysis revealed a dynamic and genotype-dependent remodeling of the Akt/mTOR signaling axis across the three experimental time points.

At 4 days post-denervation (Figure 5B and Fig. S8A–D), Pik3ip1 protein levels were increased in denervated muscles in all genotypes and appeared more elevated in mD2KO and TRKO muscles compared with CTR. In agreement with enhanced Pik3ip1 expression, denervated muscles were associated with an overall reduction in Akt/mTOR pathway activity, as indicated by decreased phosphorylation of Akt (Ser473) and S6K (Thr389), particularly in mutant muscles. Changes in mTOR phosphorylation were more variable but generally consistent with an impaired anabolic signaling response under conditions of defective TH signaling.

At 10 days post-denervation (Figure 5C and Fig. S9A–D), Pik3ip1 levels progressively declined compared with the early injury stage, although remained comparatively elevated in mD2KO and TRKO muscles relative to CTR. Concurrently, mD2KO and TRKO muscles displayed a weaker activation of downstream Akt/mTOR signaling components, suggesting an impaired anabolic response under conditions of defective TH signaling.

By 14 days post-denervation (Figure 5D and Fig. S10A–D), Pik3ip1 levels further declined, although remaining comparatively higher in mutant muscles. CTR muscles showed an overall attenuation of Akt/mTOR pathway activation, with weak phosphorylation of Akt, mTOR, and S6K, potentially reflecting an earlier adaptation to denervation. In contrast, mD2KO and TRKO muscles retained elevated phosphorylation of Akt (Ser473), mTOR (Ser2448), and S6K (Thr389), consistent with a prolonged remodeling response under impaired TH signaling conditions.

Together, these findings suggest that Pik3ip1 emerges as a potential early mediator of the altered PI3K/Akt response observed in TH-deficient denervated muscle, potentially contributing to the impaired adaptive signaling and subsequent anabolic-catabolic imbalance.

## 4 Discussion

Skeletal muscle atrophy is a tightly regulated process governed by the interplay of anabolic and catabolic pathways and is profoundly influenced by systemic endocrine signals. Hormonal cues, including glucocorticoids, insulin, and Thyroid Hormones (THs), play a central role in modulating muscle mass by coordinating metabolic adaptation, protein turnover, and cellular stress responses [21,34,61,62]. Notably, THs can exert both anabolic and catabolic effects on skeletal muscle, promoting either hypertrophy or atrophy depending on the physiological or pathological context [4,5,63,64]. This duality underscores the importance of finely tuned TH homeostasis in maintaining the balance between atrophic and hypertrophic signaling pathways.

Despite this well-established role, the molecular mechanisms linking TH signaling to the control of anabolic pathways during muscle atrophy remain incompletely understood. In addition, although THs are known to stimulate Akt phosphorylation and activate the PI3K/Akt/mTOR pathway in skeletal muscle, the molecular mechanisms linking TH signaling to this signaling remain largely unknown. In this study, we identify a previously unrecognized mechanism by which TH signaling regulates muscle mass through the control of the PI3K/Akt/mTOR pathway via Pik3ip1.

A key observation emerging from our transcriptomic analysis is the identification of a conserved gene expression signature associated with impaired TH signaling, shared between mD2KO and TRKO models. Within this program, Pik3ip1 represents one of the most consistently upregulated genes, suggesting that it functions as a critical node integrating TH-dependent transcriptional regulation with anabolic signaling pathways. The convergence of D2-dependent T3 production and TR-mediated transcription on Pik3ip1 expression indicates that both intracellular hormone availability and receptor signaling are required to maintain proper regulation of this pathway. Although *in silico* promoter analysis identified several putative TREs within the Pik3ip1 regulatory region, these predictions were not experimentally validated in the present study. Therefore, direct transcriptional regulation of Pik3ip1 by TRs remains to be formally demonstrated, and future studies employing chromatin immunoprecipitation and promoter-reporter approaches will be required to confirm functional receptor binding.

These data are in line with previous demonstration that the TH receptor β (TRβ) suppresses PI3K in follicular thyroid cancer and breast cancer by binding to the PI3K regulatory subunit p85α [62,65,66]. In addition, Davidson and colleagues reported that TRβ suppresses PI3K signaling in Anaplastic Thyroid Cancer (ATC) cell lines through unreported genomic mechanisms, including a decrease in Receptor Tyrosine Kinases (RTK) expression and an increase in phosphoinositide and Akt phosphatase expression [67]. These findings add a further mechanism by which THs regulate the Akt signal transduction.

Mechanistically, our data confirm that Pik3ip1 acts as a TH-dependent negative regulator of the PI3K/Akt/mTOR axis in skeletal muscle. Overexpression studies in C2C12 myotubes show that Pik3ip1 is sufficient to suppress Akt and mTOR activation, providing direct functional evidence that its induction can impair anabolic signaling. These findings are consistent with the known role of Pik3ip1 as a PI3K-interacting inhibitor and extend its function to skeletal muscle, where it emerges as a key regulator of the anabolic–catabolic balance [29,30,32]. Our findings are also consistent with previous evidence supporting a role for Pik3ip1 in muscle biology. Li and colleagues demonstrated that modulation of the PI3K/Akt/mTOR pathway is required for myogenic differentiation and identified Pik3ip1 among regulators contributing to this process, highlighting the importance of tightly controlled PI3K signaling during muscle maturation [33]. Moreover, transcriptomic analyses performed in models of androgen-dependent muscle atrophy identified Pik3ip1 among genes associated with the atrophic response, suggesting that increased Pik3ip1 expression may represent a common molecular feature of muscle wasting conditions [51]. The present study extends these observations by identifying TH signaling as an upstream endocrine mechanism controlling Pik3ip1 expression and by linking sustained Pik3ip1 induction to defective anabolic adaptation during denervation-induced muscle atrophy.

Importantly, we show that Pik3ip1 is dynamically regulated during denervation-induced muscle atrophy, with a rapid induction at early stages of muscle remodeling. This temporal pattern suggests that Pik3ip1 may function as an early sensor or mediator of catabolic signaling during atrophic stress. Notably, in mD2KO and TRKO mice, Pik3ip1 expression remains persistently elevated, indicating a failure to properly resolve this early response. This sustained expression is associated with impaired activation of the Akt/mTOR pathway and exacerbated muscle wasting, suggesting that defective TH signaling disrupts the temporal coordination of anabolic adaptation during denervation. Interestingly, this pattern is consistent with several features classically associated with atrophy-related genes (atrogenes), namely induction during catabolic stress, persistence in conditions of enhanced muscle wasting, and functional association with pathways controlling muscle mass [68]. However, further studies involving direct *in vivo* manipulation of Pik3ip1 will be required to determine whether it fulfills the criteria of a bona fide atrogene.

These observations support a model in which TH signaling is required not only to activate metabolic and regenerative programs, but also to suppress inappropriate or prolonged catabolic responses. In physiological conditions, transient induction of Pik3ip1 may contribute to the initial adaptation to denervation by restraining anabolic signaling. However, timely repression of Pik3ip1 appears necessary to restore Akt/mTOR activity and support muscle recovery. In the absence of adequate TH signaling, this repression fails to occur, leading to sustained inhibition of anabolic pathways and progressive muscle atrophy. The identification of Pik3ip1 as a TH-responsive gene also provides insight into the broader role of TH signaling in muscle homeostasis. Previous studies have primarily focused on the ability of TH to promote mitochondrial function, oxidative metabolism, and myogenic differentiation [4,7]. Our data expand this view by demonstrating that TH signaling also directly modulates intracellular signaling pathways controlling protein synthesis and degradation. In this context, the TH-Pik3ip1 axis represents a previously unrecognized mechanism linking endocrine regulation to the control of PI3K/Akt/mTOR activity.

From a translational perspective, these findings have important implications. Muscle atrophy is a common feature of a wide range of pathological conditions, including denervation, aging, chronic disease, and endocrine disorders [69]. The identification of Pik3ip1 as a mediator of TH-dependent anabolic control highlights its potential relevance in the regulation of muscle mass. Although the present findings are limited to experimental models, they suggest that Pik3ip1 may represent a previously unrecognized component of the transcriptional program associated with muscle wasting. Future studies employing direct *in vivo* manipulation of Pik3ip1 will be required to determine its functional contribution to muscle atrophy and its potential translational relevance. In particular, restoring TH signaling or directly targeting Pik3ip1 could potentially improve anabolic responsiveness in conditions characterized by impaired Akt/mTOR activation.

Interestingly, increased Pik3ip1 expression has also been proposed as a urinary biomarker associated with exertional rhabdomyolysis and muscle injury in humans [70]. Although the biological significance of this observation remains unclear, it raises the possibility that Pik3ip1 may reflect broader alterations in muscle homeostasis beyond experimental models of atrophy. Future studies will be required to determine whether the TH-Pik3ip1 axis contributes to muscle damage responses and muscle wasting across additional pathological settings.

In conclusion, our findings identify Pik3ip1 as a key mediator of TH-dependent regulation of skeletal muscle homeostasis and establish the TH/Pik3ip1/PI3K/Akt/mTOR axis as a critical pathway controlling muscle adaptation to atrophic stress. By linking endocrine signaling to intracellular anabolic pathways, this work provides a new conceptual framework for understanding the regulation of muscle mass and suggests novel opportunities for therapeutic intervention in muscle wasting conditions.

## 5 Limitation of the study

Several limitations of the present study should be acknowledged. First, both the mD2KO and TRKO models rely on constitutive genetic manipulations, and therefore developmental effects on skeletal muscle physiology cannot be completely excluded. In particular, TRKO mice exhibit a well-established developmental and metabolic skeletal muscle phenotype characterized by reduced muscle size and altered muscle homeostasis. Nevertheless, the inclusion of the TRKO model was intended to assess the requirement of receptor-mediated TH signaling downstream of T3 production, and the convergence of mD2KO and TRKO muscles on a common transcriptional, signaling, and phenotypic response supports the interpretation that the observed effects are primarily linked to impaired TH signaling rather than to model-specific developmental alterations. Future studies employing inducible genetic models will be required to fully distinguish developmental from adaptive effects.

Second, while Pik3ip1 emerged as one of the most consistently deregulated genes across all experimental systems examined and functional studies demonstrated that its overexpression is sufficient to suppress Akt/mTOR signaling *in vitro*, its causal contribution to muscle remodeling has not yet been directly established *in vivo*. Therefore, the necessity and sufficiency of Pik3ip1 for denervation-induced muscle atrophy remain to be formally demonstrated using muscle-specific gain- and loss-of-function approaches.

## CRediT authorship contribution statement

**Annarita Nappi:** Writing – original draft, Validation, Software, Methodology, Investigation, Formal analysis, Data curation, Conceptualization. **Serena Sagliocchi:** Methodology, Investigation. **Federica Restolfer:** Methodology, Investigation. **Caterina Miro:** Methodology, Investigation. **Lucia Acampora:** Methodology, Investigation. **Giovanna Giuseppina Altobelli:** Methodology, Investigation. **Monica Dentice:** Writing – original draft, Supervision, Data curation, Conceptualization. **Annunziata Gaetana Cicatiello:** Writing – original draft, Supervision, Data curation, Conceptualization.

## Ethical statement

All animal procedures were conducted in accordance with national and European regulations for the care and use of laboratory animals and were approved by the Italian Ministry of Health and the local Institutional Animal Care and Use Committee (IACUC). The approved protocols include Project number 354/2019-PR (Protocol number D5A89.38), Project number 1/2024-PR (Protocol number D5A89.73), and Project number 757/2025-PR (Protocol number D5A89.105).

## Use of generative AI

The authors declare that no Generative Artificial Intelligence (AI) tools or AI-assisted technologies were used during the preparation of this manuscript.

## Funding

This work was supported by grants from 10.13039/100020581AIRC Foundation for Cancer Research in Italy to M.D. (IG 29242) and to C.M. (MFAG 30433).

## Declaration of competing interest

The authors declare that they have no known competing interests or personal relationships that could have appeared to influence the work reported in this paper.

## Data Availability

Data will be made available on request.
