# Supplementary figures and images for: Pik3ip1 mediates thyroid hormone-dependent regulation of the PI3K/Akt/mTOR axis in muscle atrophy
Source: Mol Metab. 2026 Jul 6;111:102413. doi: 10.1016/j.molmet.2026.102413 (PMC13383864; doi:10.1016/j.molmet.2026.102413)

A

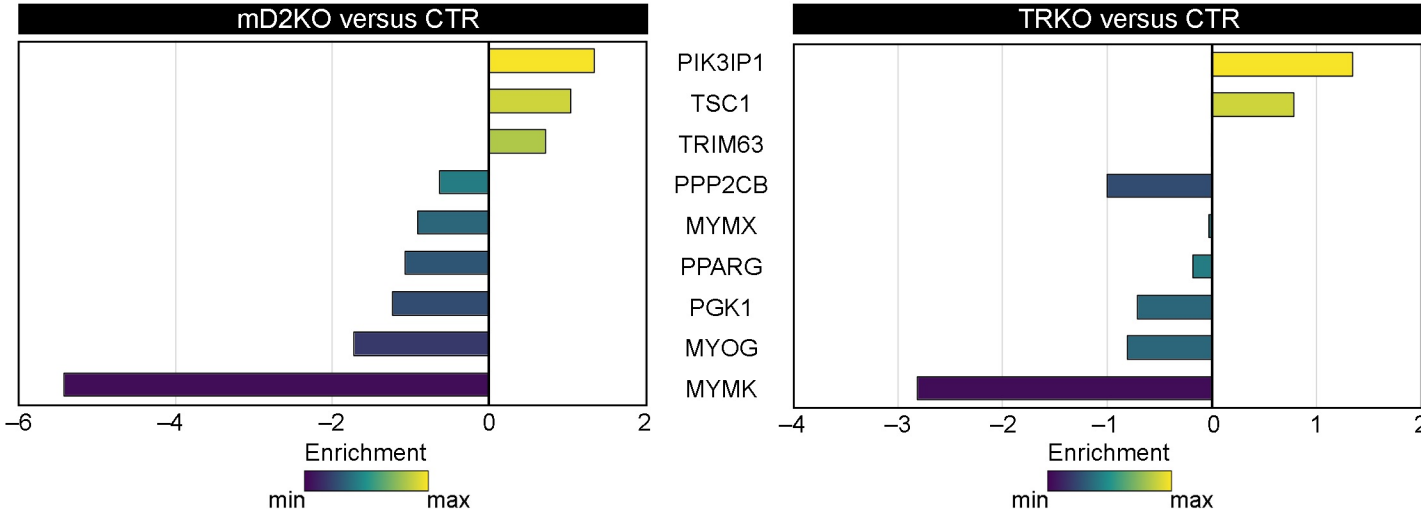

B

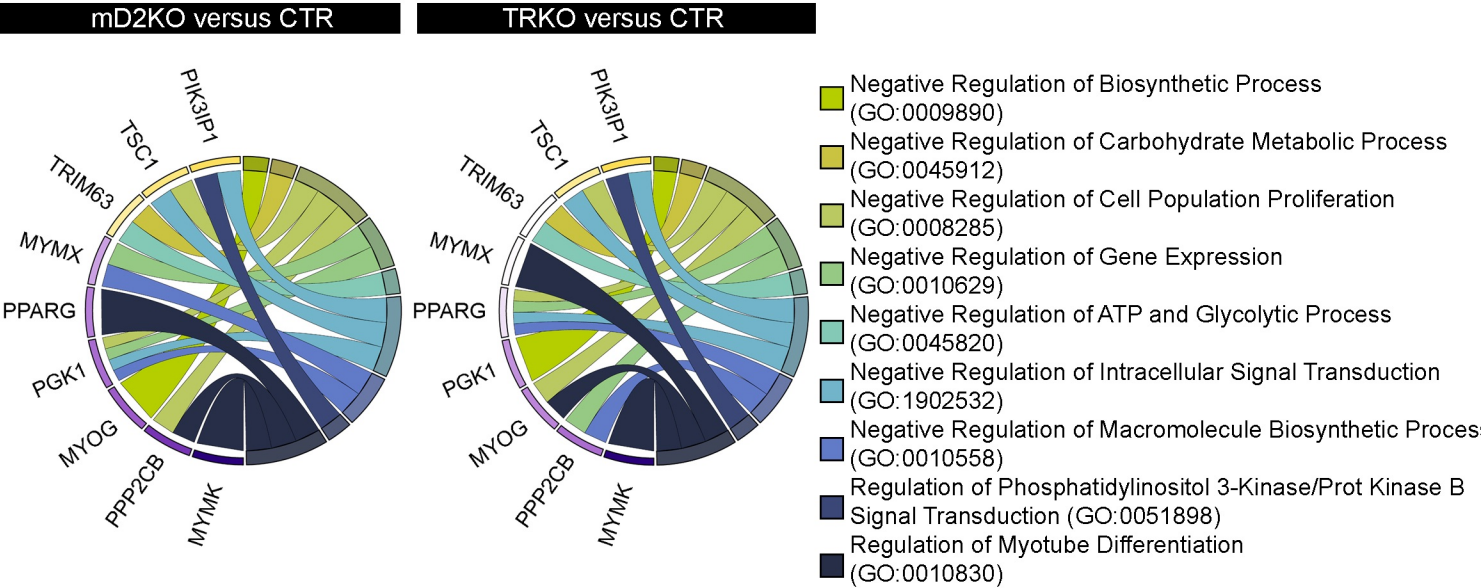

Figure S1

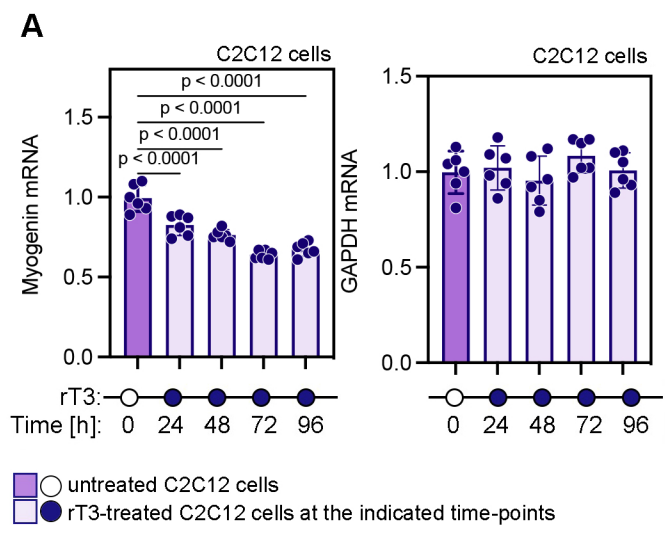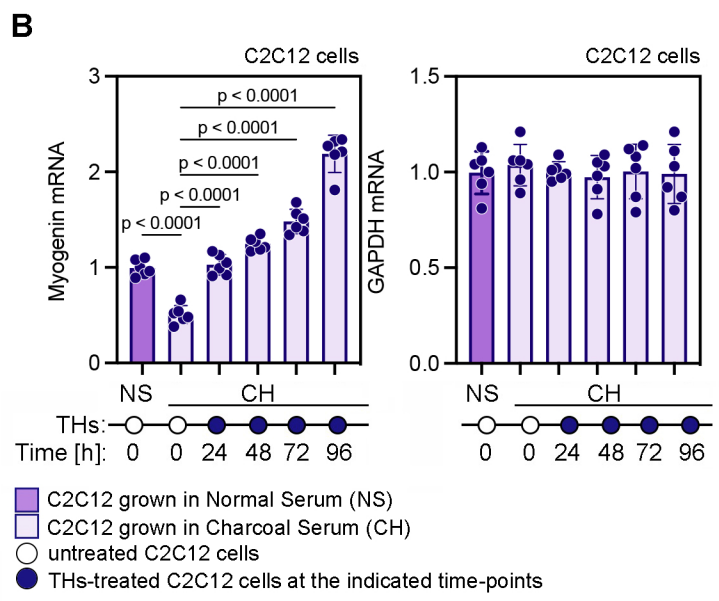

Figure S2

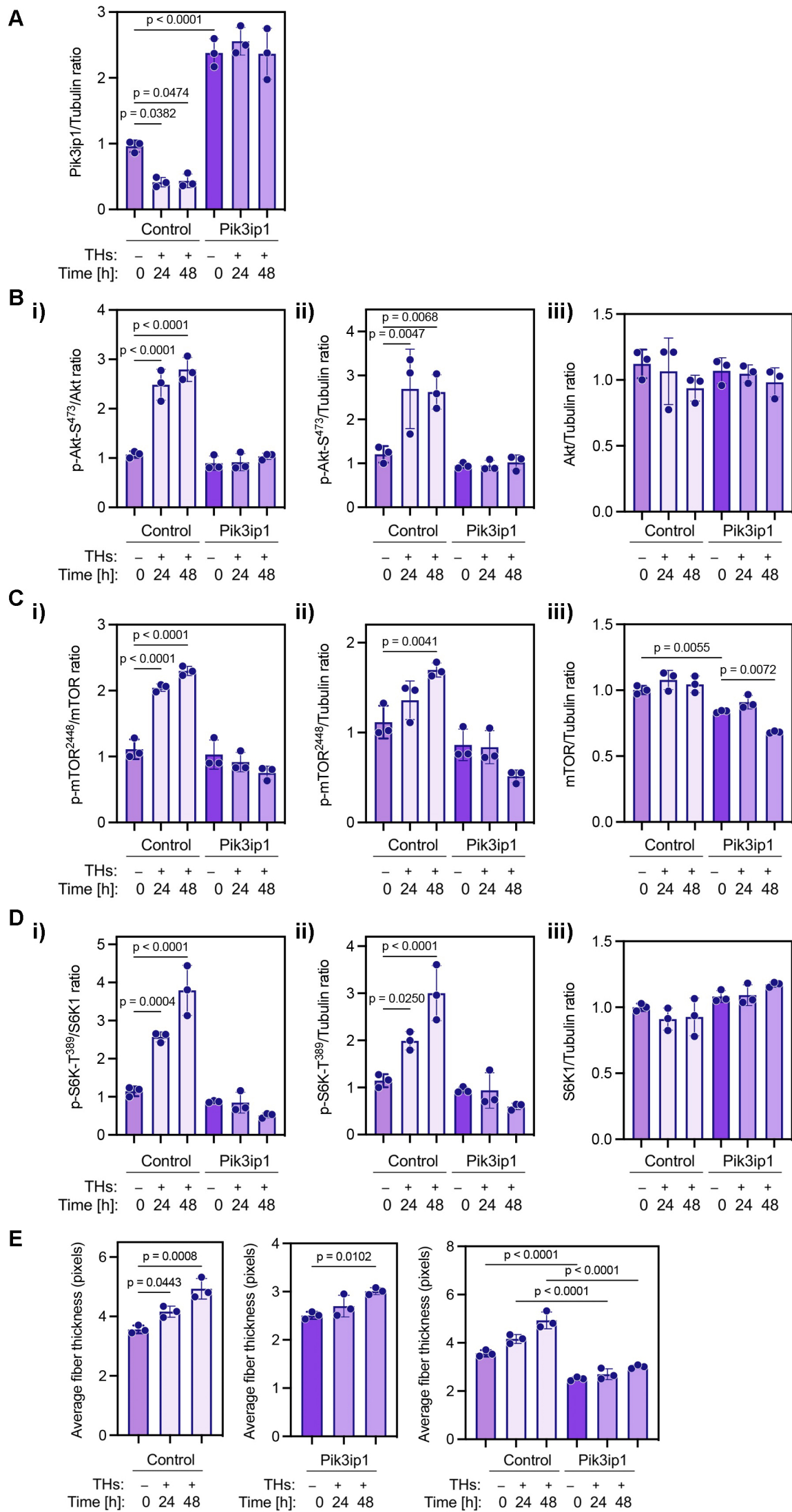

Figure S3

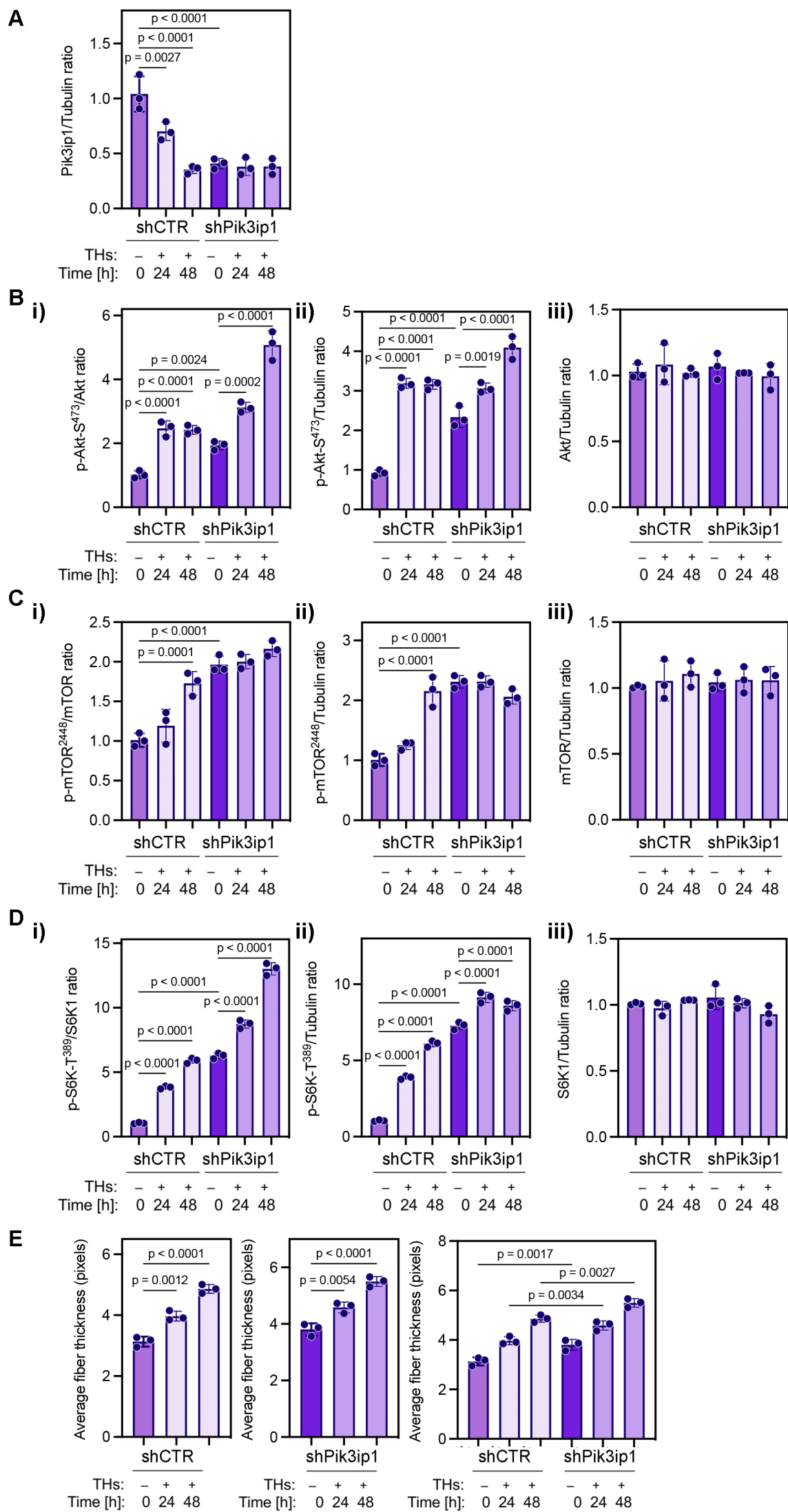

Figure S4

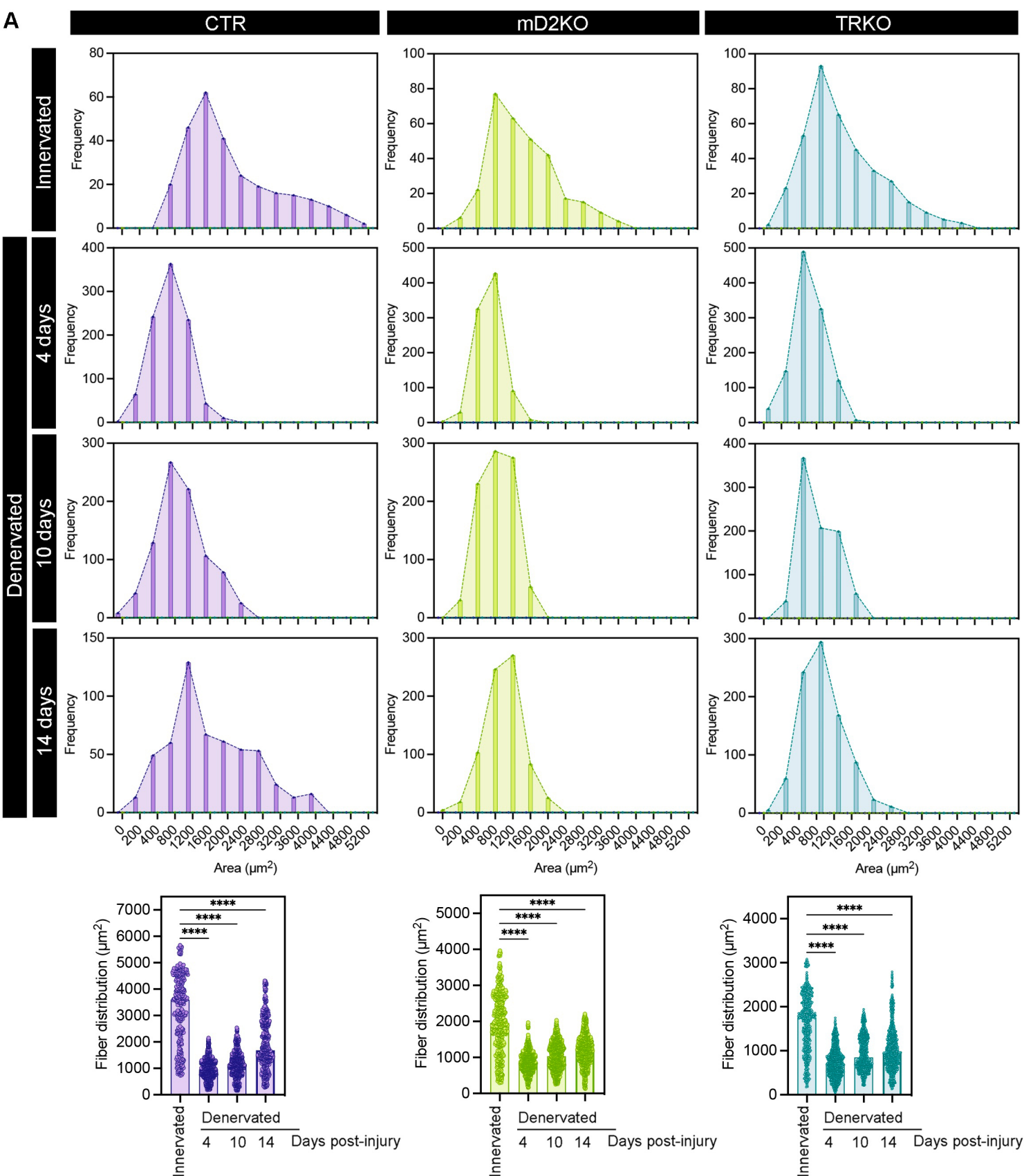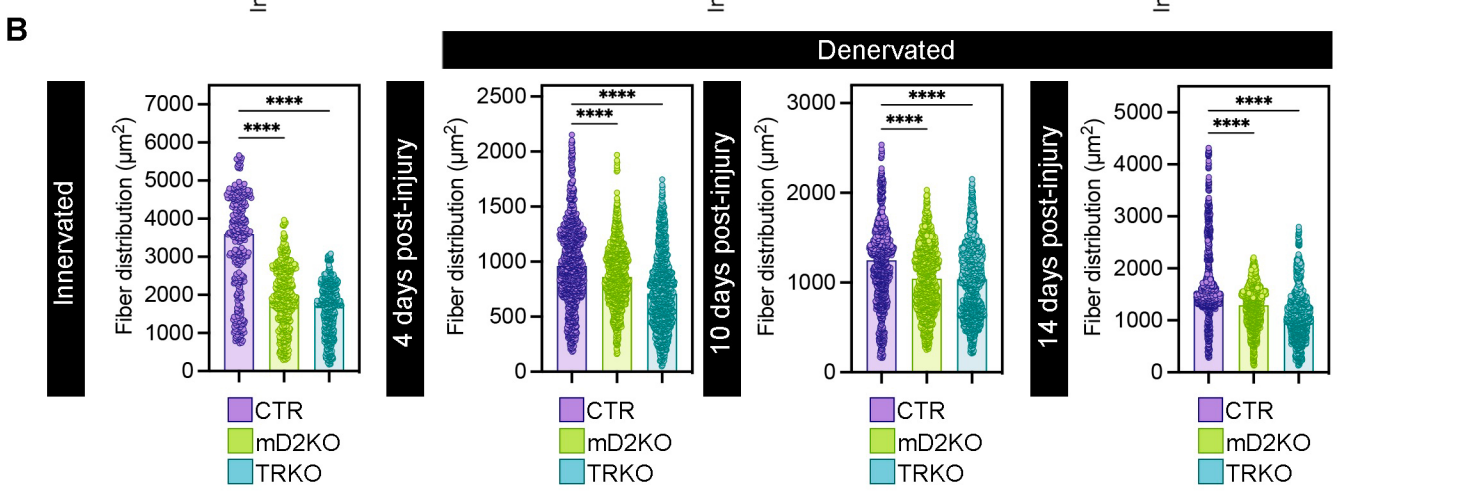

**Figure S5**

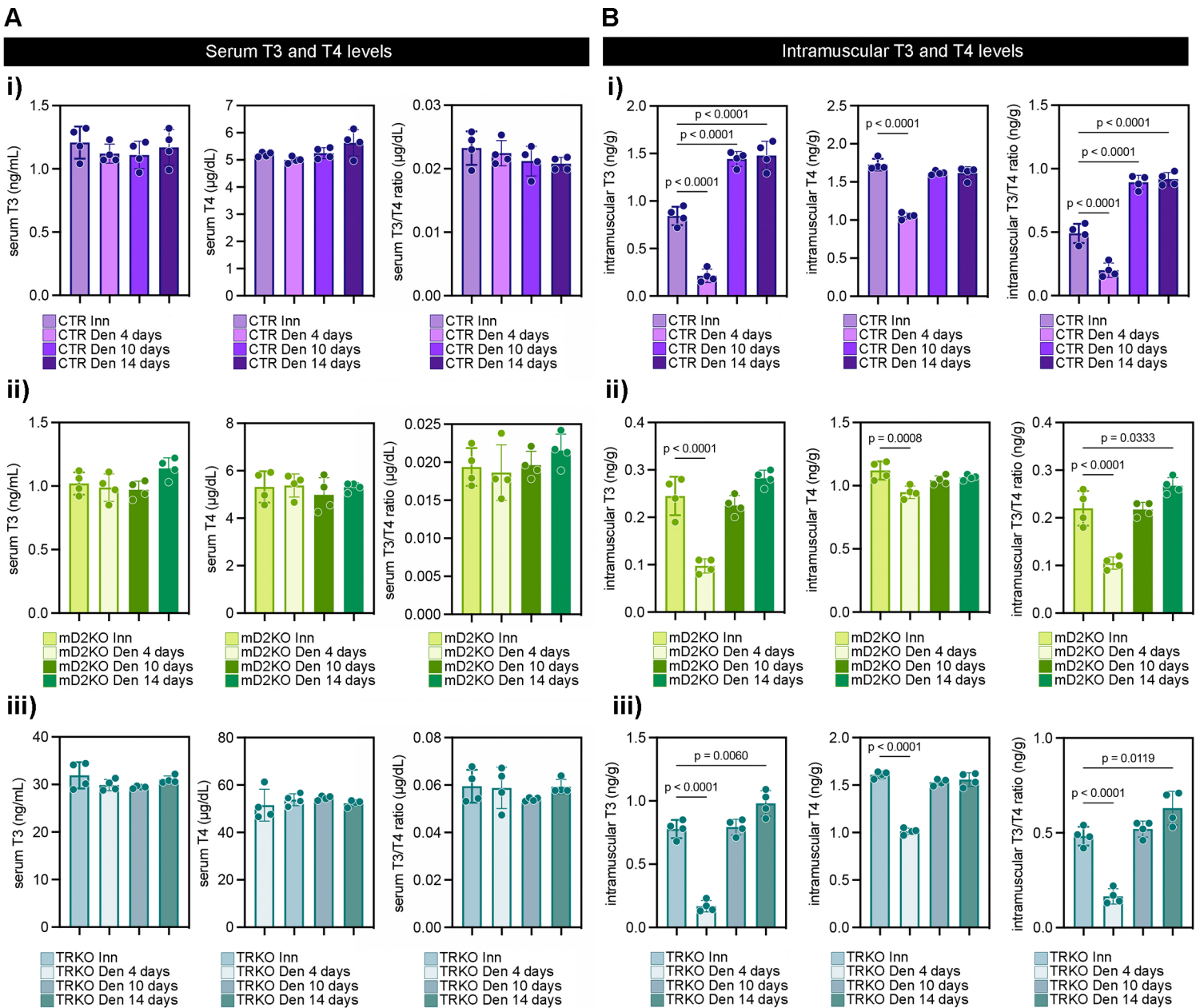

Figure S6

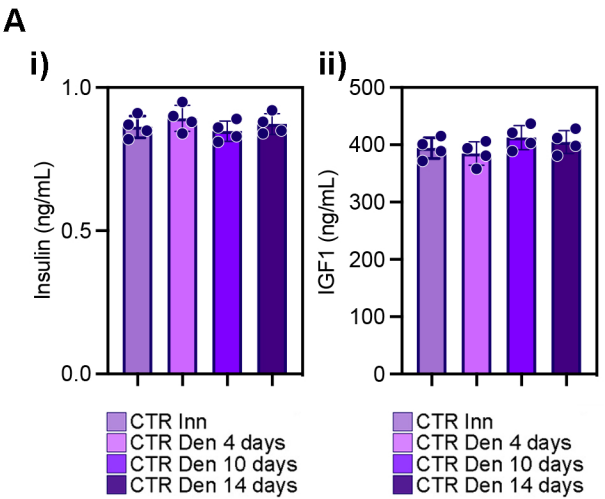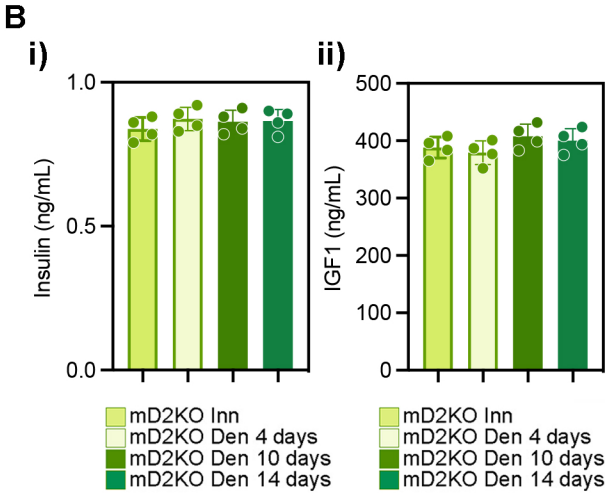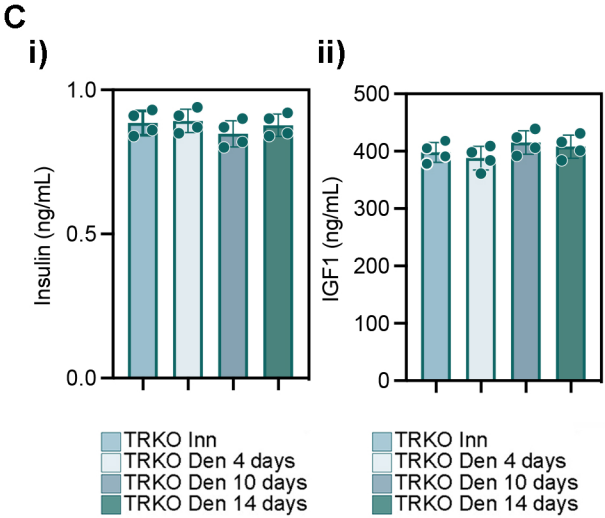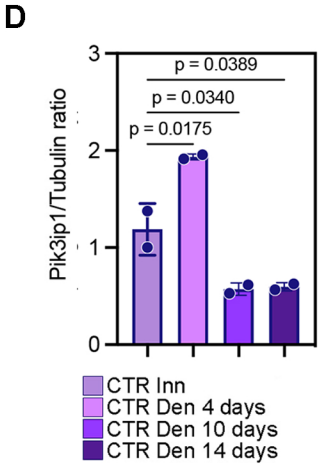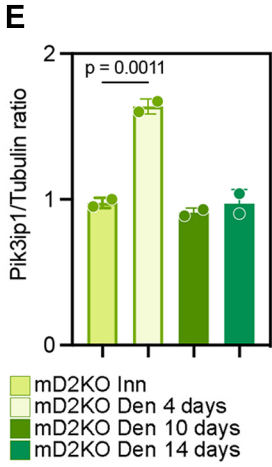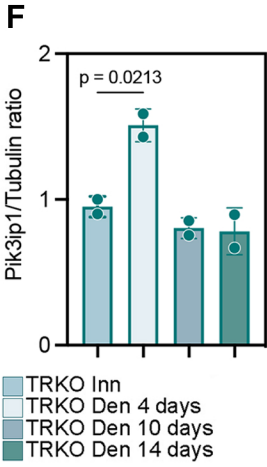

Figure S7

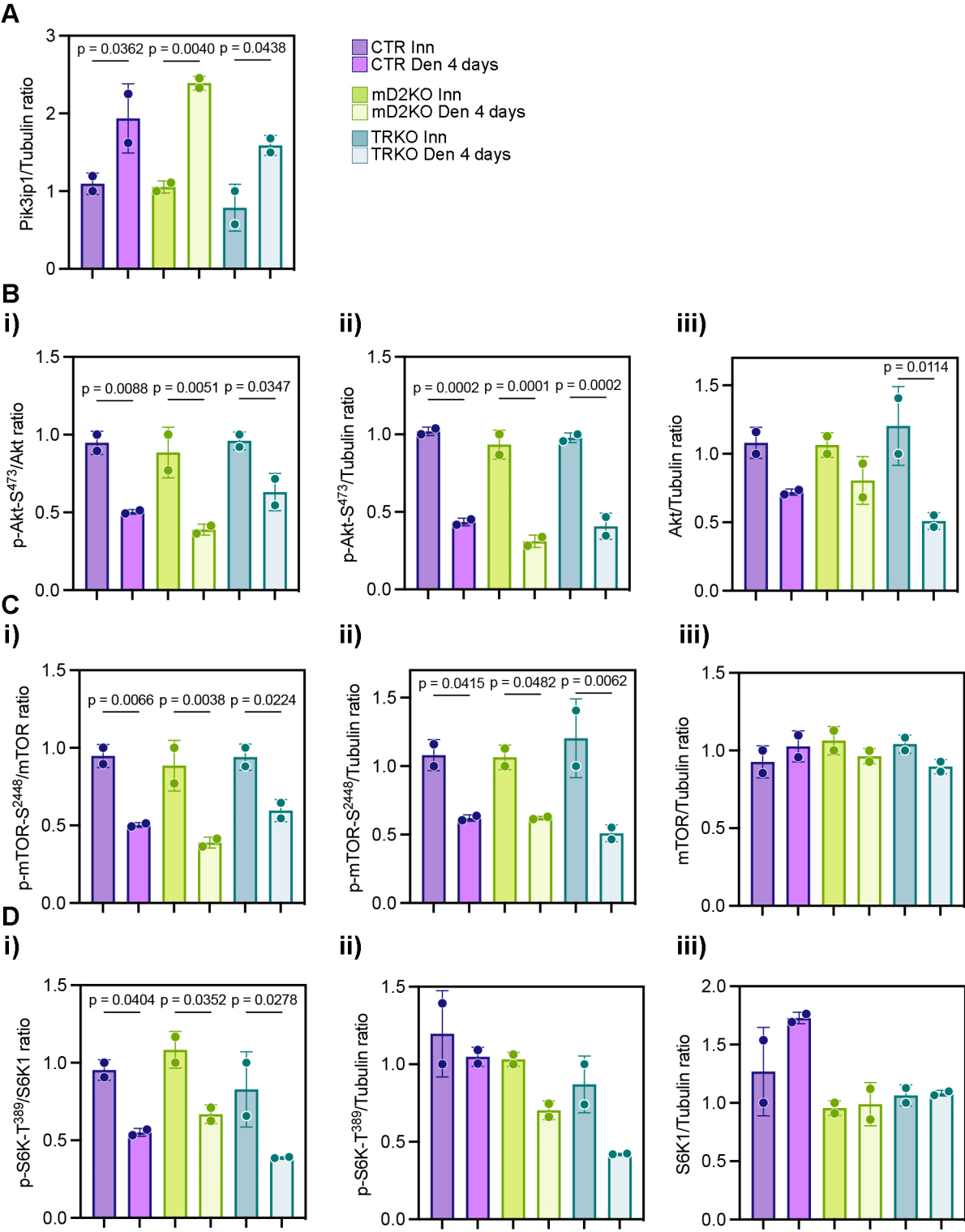

Figure S8

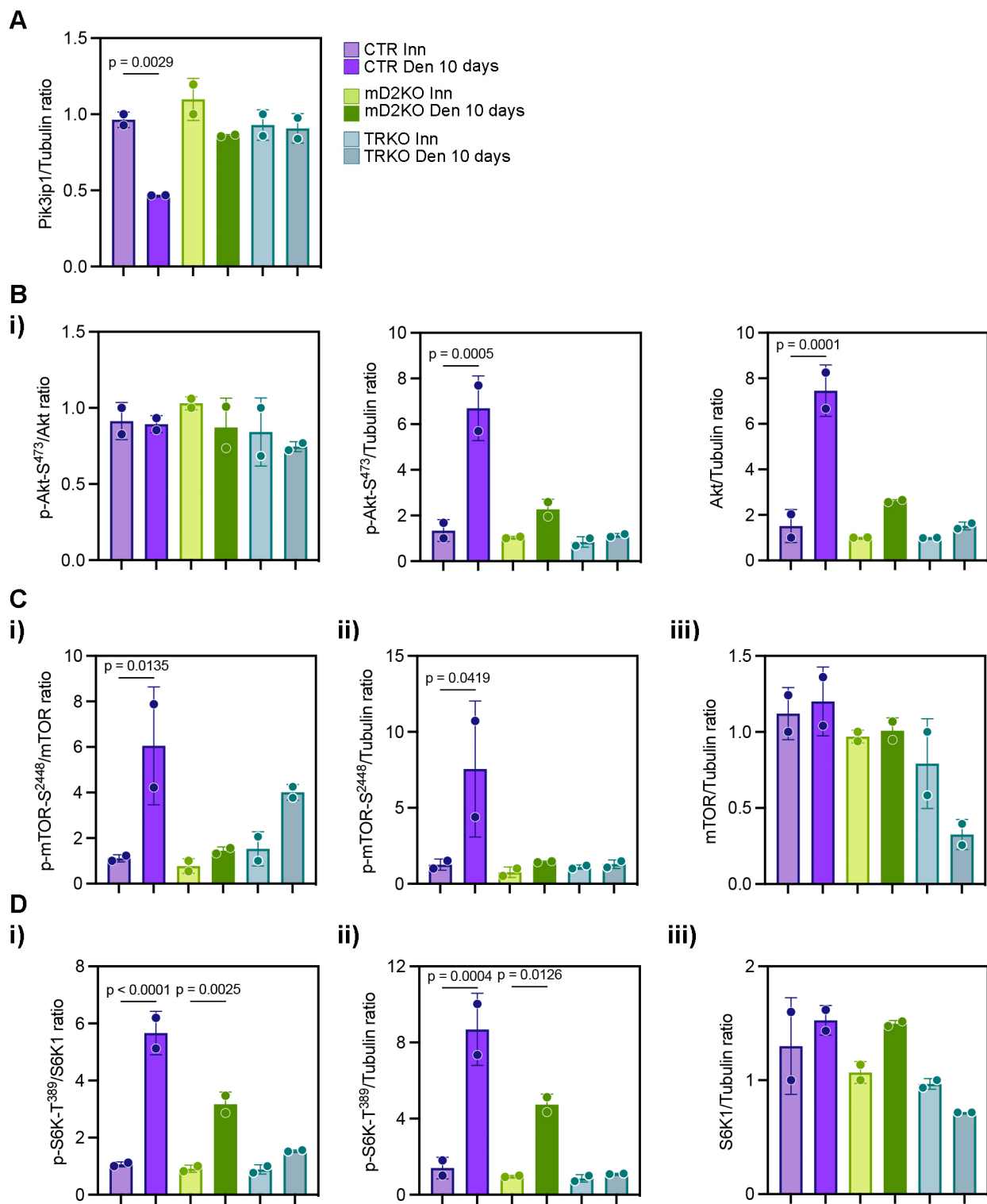

Figure S9

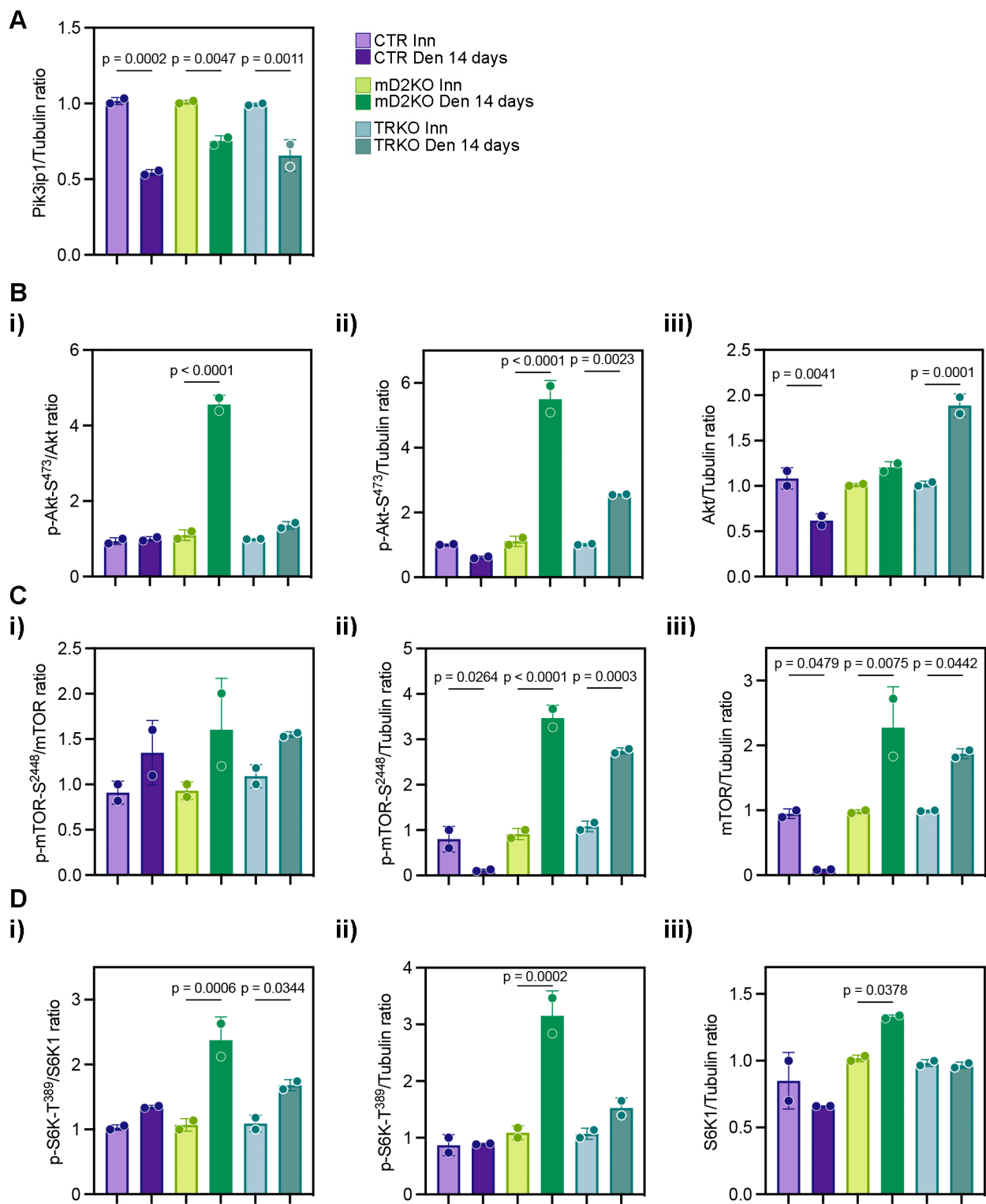

Figure S10

Supplement: Multimedia component 2 [file mmc2.pdf]
